# Synthetic robust perfect adaptation achieved by negative feedback coupling with linear weak positive feedback

**DOI:** 10.1093/nar/gkac066

**Published:** 2022-02-15

**Authors:** Zhi Sun, Weijia Wei, Mingyue Zhang, Wenjia Shi, Yeqing Zong, Yihua Chen, Xiaojing Yang, Bo Yu, Chao Tang, Chunbo Lou

**Affiliations:** CAS Key Laboratory of Microbial Physiological and Metabolic Engineering, State Key Laboratory of Mycology, Institute of Microbiology, Chinese Academy of Sciences, Beijing 100101, China; College of Life Sciences, University of Chinese Academy of Sciences, Beijing 100149, China; State Key Laboratory of Microbial Resources, Institute of Microbiology, Chinese Academy of Sciences, Beijing 100101, China; College of Life Sciences, University of Chinese Academy of Sciences, Beijing 100149, China; Center for Quantitative Biology, Peking-Tsinghua Center for Life Sciences, Academy for Advanced Interdisciplinary Studies, Peking University, Beijing100871, China; School of Physics, Peking University, Beijing 100871, China; Department of Applied Physics, School of Sciences, Xi’an University of Technology, Xi’an 710048, China; Bluepha Co., Ltd, Beijing 102206, China; State Key Laboratory of Microbial Resources, Institute of Microbiology, Chinese Academy of Sciences, Beijing 100101, China; College of Life Sciences, University of Chinese Academy of Sciences, Beijing 100149, China; Center for Quantitative Biology, Peking-Tsinghua Center for Life Sciences, Academy for Advanced Interdisciplinary Studies, Peking University, Beijing100871, China; CAS Key Laboratory of Microbial Physiological and Metabolic Engineering, State Key Laboratory of Mycology, Institute of Microbiology, Chinese Academy of Sciences, Beijing 100101, China; Center for Quantitative Biology, Peking-Tsinghua Center for Life Sciences, Academy for Advanced Interdisciplinary Studies, Peking University, Beijing100871, China; School of Physics, Peking University, Beijing 100871, China; Center for Cell and Gene Circuit Design, CAS Key Laboratory of Quantitative Engineering Biology, Guangdong Provincial Key Laboratory of Synthetic Genomics, Shenzhen Key Laboratory of Synthetic Genomics, Shenzhen Institute of Synthetic Biology, Shenzhen Institutes of Advanced Technology, Chinese Academy of Sciences, Shenzhen 518055, China; College of Life Sciences, University of Chinese Academy of Sciences, Beijing 100149, China

## Abstract

Unlike their natural counterparts, synthetic genetic circuits are usually fragile in the face of environmental perturbations and genetic mutations. Several theoretical robust genetic circuits have been designed, but their performance under real-world conditions has not yet been carefully evaluated. Here, we designed and synthesized a new robust perfect adaptation circuit composed of two-node negative feedback coupling with linear positive feedback on the buffer node. As a key feature, the linear positive feedback was fine-tuned to evaluate its necessity. We found that the desired function was robustly achieved when genetic parameters were varied by systematically perturbing all interacting parts within the topology, and the necessity of the completeness of the topological structures was evaluated by destroying key circuit features. Furthermore, different environmental perturbances were imposed onto the circuit by changing growth rates, carbon metabolic strategies and even chassis cells, and the designed perfect adaptation function was still achieved under all conditions. The successful design of a robust perfect adaptation circuit indicated that the top-down design strategy is capable of predictably guiding bottom-up engineering for robust genetic circuits. This robust adaptation circuit could be integrated as a motif into more complex circuits to robustly implement more sophisticated and critical biological functions.

## INTRODUCTION

Biological systems are constantly subjected to genetic variations and environmental fluctuations (1,2). They employ a number of strategies to increase robustness to cope with internal and external disturbances, including gene redundancy, network topology design, and distributed regulatory networks (3–6). Several regulatory motifs were identified from complex genetic circuits by systematic theoretical analysis to execute robust biological functions, such as adaptation (7,8), fold-change detection (9), symmetry breaking (10) and switch-like behaviors (11). Discovering and analyzing the functions and behaviors of such motifs has become an important part of the development of systems and synthetic biology in the last two decades (12–15).

**Figure 1.**
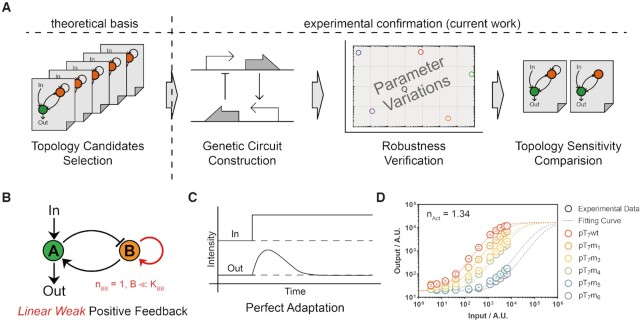
Scheme of the topology-based design strategy for a robust perfect adaptation (RPA) circuit. (**A**) Graphic workflow summary of this study. (**B**) The chosen topology and key constraint for the robust perfect adaptation circuit. (**C**) The expected dynamic behavior of an adaptation circuit. (**D**) Monomeric transcriptional activator and activation curves with different promoter mutants (red: pT_7_wt, orange: pT_7_m_1_, yellow: pT_7_m_3_, green: pT_7_m_4_, blue: pT_7_m_5_, gray: pT_7_m_6_). The points are the mean values of steady-state input–output data, and the error bar indicates the standard deviation (S.D.) of three independent replicates (*n* = 3). The curves are fitting results using the activation Hill function from our previous works (38).

Biologically, perfect adaptation, in some cases known as homeostasis, is an important cellular function for maintaining the ability to respond to changing external stimulation while subsequently returning to an almost unperturbed level of internal components (Figure 1C) (16,17); several adaptation circuit motifs have been identified. Synthetic robust perfect adaptation (RPA), as a high-performance adaptation function, is in high demand in synthetic biology (18,19). Unfortunately, previous synthetic genetic circuits are notoriously fragile when exposed to internal and external environmental disturbances, and they can easily lose functioning when transferring from the laboratory to real-world medical or industrial situations (20–25). Numerous theoretical robust circuits have been designed, but few of them have experimentally evaluated robustness when facing perturbations (9,11,26–29). In the field of cybernetics and robotization, a common strategy is to embed a feedback controller module, such as a PID Controller, to set the output of interest to a target point, thus increasing the robustness of the system function when facing environmental fluctuations (30–32). Several recent works have implemented these feedback control strategies in synthetic biological system design and successfully realized a series of robust homeostasis functions (33–37).

Here, we report the experimental design and construction of an RPA circuit that incorporates 2-node negative feedback coupling with linear positive feedback on the buffer node. Our previous work showed that a negative feedback circuit for transcriptional regulation must incorporate additional positive feedback on the buffer node to achieve a perfect adaptation function. More intriguingly, the linearity of the positive feedback could endow the circuit with the ability to robustly withstand environmental and genetic perturbations (8). In this work, we first re-evaluated the linear activation ability of an activator part (38) and then incorporated negative feedback with weak linear positive feedback to create a combined RPA circuit (Figure 1A). The robustness of the circuit was measured by changing specific genetic parts and deleting each feedback interaction. Furthermore, several strategies involving global-parameter perturbations were tested, and perfect adaptations were successfully achieved. Our results highlighted a novel topology and the importance of linear positive feedback in achieving perfect adaptation robustly. The principles used here to design and test robust circuits could facilitate the transition of proof-of-concept circuits to real-world biocybernetic applications, especially when dealing with uncertain or fluctuating environmental conditions, thus releasing the tremendous power of both modern control theory and molecular genetic biology.

## MATERIALS AND METHODS

### Strain, media and chemicals

*Escherichia coli* K-12 sub strain DH10B (F^–^*mcrA* Δ(*mrr-hsdRMS-mcrBC*) φ80*lacZ*ΔM15 Δ*lacX74 recA1 araD139*Δ(*ara-leu*)7697 *galU galK rpsL*(Str^R^) *endA1 nupG*) was used for all cloning and testing experiments. For performing cross-species and cross-genus tests, other *E. coli* strains, including BW25113 (Δ(*araD-araB*)567 Δ(*rhaD-rhaB*)568 Δ*lacZ4787* (::rrnB-3) *hsdR514 rph-1*) and BL21 (*E. coli* B F^–^*ompT gal dcm lon hsdS_B_*(r_B_^–^ m_B_^–^) [*malB*^+^]_K-12_(λ^S^)), and an environmentally tolerant strain, *Pseudomonas putida* KT2440, were used (39–41). Cells were grown in either LB medium (10 g/l tryptone, 5 g/l yeast extract, and 10 g/l NaCl) or M9 medium containing M9 minimal salts (6.78 g/l Na_2_HPO_4_, 3 g/l KH_2_PO_4_, 1 g/l NH_4_Cl, 0.5 g/l NaCl), 0.4% (m/v) D-glucose or D-fructose, or a mixed carbon source of 20 mM succinate and 15 mM pyruvate, 0.2% (m/v) casamino acids (BD Bacto, 223120), 0.34 g/l thiamine hydrochloride, 2 mM MgSO_4_ and 0.1 mM CaCl_2_. For the agar plates, 15 g/l agar was added. Antibiotics used to select and maintain plasmids included 100 μg/ml ampicillin, 50 μg/ml kanamycin, 100 μg/ml apramycin and 25 μg/ml irgasan. The chemical inducers were isopropyl β-D-1-thiogalactopyranoside (IPTG) and 4-isopropyl benzoic acid (Cumate). All chemicals used in the study were purchased from Sigma–Aldrich unless stated otherwise.

### Detailed plasmid specifications

For characterization of transcriptional activation of T_7_ RNAP, integrated *E. coli* DH10B strains were used as previously described (38). An pTac-T_7_ RNAP (for output test) or pTac-sfGFP (for input test) cassette was integrated into the chromosome using pOSIP. For the output test, several different T_7_ promoter mutants for the reporter cassette were used; the sequence details are summarized in Supplementary Table S4.

**Figure 2.**
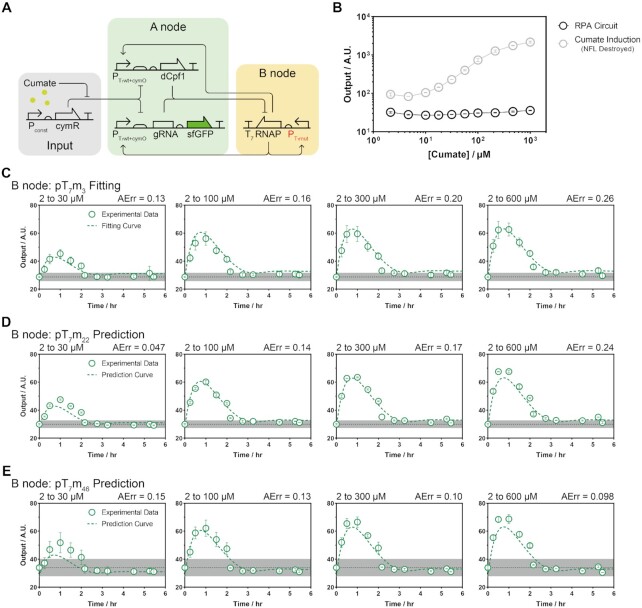
Genetic circuit construction and characterization. (**A**) Scheme of the RPA circuit composed of genetic regulatory components according to the designed topology. Substituted parts for fitting and predicting are indicated in red. (**B**) The steady-state output of the RPA circuit and the simple input–output system under different constant concentrations of input inducer. The circuit of the simple input–output system is shown in Supplementary Figure S2A. The points are the mean of three biological replicates (*n* = 3), and the error bar indicates the standard deviation (S.D.). (**C–E**) Time-course curves for switching into several final input signals for three different positive feedback activities on node B. The step-like switching time point was set as the initial time. The initial and final signal concentrations and the defined AErr indexes are shown in each subfigure. The colored points are the mean of three biological replicates (*n* = 3), and the error bar indicates the S.D.. Curves in (C) are the fitted results for the experimental data, and those in (D) & (E) are the predictions. Other curves with different final input concentrations are summarized in Supplementary Figure S3. The dark dotted lines are the mean fluctuating non-switched output signals, and the gray shaded regions are the S.D. of all the recorded data.

For construction of the RPA circuit, three plasmids were used as basic backbones to carry the A node (pAR & pAP) and B node (pBB). The plasmid details are summarized in Supplementary Table S1. Among all the circuits tested, we defined the first mentioned circuit construction as the initial version (RPA v1.0, Figure2B, C). The circuit construction details are summarized in Supplementary Table S2.

For characterization of the parameter perturbation behaviors, the genetic parts in the RPA v1.0 circuit were replaced with other homologous parts. The replacement details are summarized in Supplementary Table S3. For characterization of the single edges, the subcircuits were reconstructed from RPA v1.0, and the reconstruction details are summarized in Supplementary Table S4.

### Steady-state and time-course behavior characterization

All incubations were carried out using a Digital Thermostatic Shaker (AOSHENG) maintained at 37°C and 1000 rpm using Corning flat-bottom 96-well plates sealed with sealing film (Corning, BF-400-S), except for the global perturbation experiments. A previously developed quantitative method was used to characterize the steady-state behaviors of the parts and circuits (e.g. Figures 1D and 2B) (38,42). Briefly, bacteria harboring the parts or circuits of interest were first inoculated from single colonies into a 96-well plate overnight for growth in LB medium, after which the cell cultures were diluted 196-fold with M9 medium. After 3 h of growth, the cultures were further diluted 700-fold with M9 medium containing specific concentrations of inducers as needed and incubated for another 6 h. Finally, 20 μl samples of each culture were transferred to a new plate containing 180 μl per well of PBS supplemented with 2 mg/ml kanamycin for cell fixation and prepared for cytometry analysis.

The quantitative method was slightly modified for characterization of the time-course behaviors (e.g. Figure 2C–E). The bacteria were cultured in medium with a certain concentration of inducer as a preinput signal overnight for growth and the first 196-fold dilution. After 3 h of growth, 20 μl cultured samples were harvested, and the cell densities (OD_600_) were recorded. This moment was set as time zero as indicated in all adaptation time-course figures, and the cultures were then diluted in a series of different folds (e.g. 20-fold, 50-fold, 100-fold, 200-fold, 500-fold, 1000-fold) with fresh M9 medium containing specific concentrations of inducers as needed. All diluted cultures were incubated and then harvested for cytometry analysis once they reached the same OD_600_ value as that at time zero, and the harvest time points were recorded.

### Bacteria incubation in turbidostat

For characterization of the time-course behaviors under slow growth rate conditions (e.g. Figure 4A), a multichannel parallel turbidostat (Efun Electronic Design company http://www.efundesign.cn/) was used for cell incubation. The turbidity of the culture was controlled to keep bacteria growing at the exponential phase throughout the culture. Bacteria harboring the circuits of interest were first inoculated from single colonies into the turbidostat overnight for growth in M9 medium with initial level Cumate. Then, the concentration of Cumate was changed, and the incubation continued. The inducer-changing moment was set as time zero to remain consistent with previous experimental settings. Sampling time points were selected appropriately, and for each time point, 20 μl samples were harvested and prepared for cytometry following the same procedure described above.

### Flow cytometry measurement and data processing

The fluorescence distribution of each sample was assayed using an LSRII flow cytometer (BD Biosciences) with appropriate channels and voltage settings; each distribution contained >20 000 events. FlowJo (TreeStar, v10.6.2) was used for processing data and exporting statistical values.

### Data analysis and modeling

To calculate the AErr values, the steady-state output signals for different input conditions were used. For output values of step-like changed input signals ($Outpu{t_1}$), the final two or three unchanged time-course data were selected. For output values of unchanged input signals ($Outpu{t_2}$), all time-course data were used. These data were used to obtain average steady-state values for $Outpu{t_1}$ and $Outpu{t_2}$ and then calculate the AErr value according to the definition in the manuscript.

All modeling steps were carried out using MATLAB R2018a. The time-series simulations were obtained by using the ‘ode45’ function, and the parameters in Eqs (1,2), describing the dynamic features of the interaction edges, were obtained by fitting using the ‘fminsearch’ function. The predictions for the promoter substitutions were obtained using the same function, except that the parameter values were retrieved from either the fitting results of the characterization data or our previous work. The parameters and units used are summarized in Supplementary Table S5 (38).

## RESULTS

Based on the in silico simulation from the transcriptional regulatory model reported in our previous study (8), our simulation revealed that positive feedback on the buffer node was indispensable for a 2-node negative feedback loop to achieve perfect adaptation (Figure 1B). The importance of the positive feedback was computationally confirmed by increasing the sampled parameter cassettes (Supplementary Figure S1). Further theoretical investigation revealed that linear weak positive feedback ($B \ll {K_{BB}}, {n_{BB}} = 1$) was sufficient to provide perfect adaptation for the 2-node negative feedback circuit based on Eqs (1) and (2) (8). Within the equations, $\alpha$ is the maximum expression rate, and $\gamma$ is the reduction coefficient of each node. We assume that all regulations can be described using Hill functions and are insulated from each other, combined with AND logic. For each regulation, ${K_{ij}}$ and ${n_{ij}}$ represent the half-maximal concentration and hill coefficient from component $i$ to component $j$, respectively. The activation from input to node A was slightly more complex because of nondirect effects, which are common in biological systems (see Supplementary materials for more detailed information). For the linear weak positive feedback and weak leaked expression, this theoretical result indicated that the circuit would be robust and insensitive to other parameters except the above two key parameters and regulatory interactions.

(1) $$\begin{eqnarray*}{\rm{\ }}\frac{{d\left[ A \right]}}{{dt}} &=& {\alpha _1}\ \left( {\frac{1}{{1 + {{\left( {\frac{{{K_{T{F_{tot}}}}}}{{1 + \left( {\frac{{\left[ {Input} \right]}}{{{K_{IR}}}}} \right)}}} \right)}^{{n_{RA}}}}}} + {\beta _{RA}}} \right)\cdot\nonumber\\ && \left( {\frac{{{{\left[ B \right]}^{{n_{BA}}}}}}{{{K_{BA}}^{{n_{BA}}} + {{\left[ B \right]}^{{n_{BA}}}}}} + {\beta _{BA}}} \right) - {\gamma _A}\left[ A \right]\end{eqnarray*}$$

(2) $$\begin{eqnarray*}{\rm{\ }}\frac{{d\left[ B \right]}}{{dt}} &=& {\alpha _2}\ \left( {\frac{1}{{1 + {{\left( {\frac{{\left[ A \right]}}{{{K_{AB}}}}} \right)}^{{n_{AB}}}}}} + {\beta _{AB}}} \right)\cdot\nonumber\\ && \left( {\frac{{{{\left[ B \right]}^{{n_{BB}}}}}}{{{K_{BB}}^{{n_{BB}}} + {{\left[ B \right]}^{{n_{BB}}}}}} + {\beta _{BB}}} \right) - {\gamma _B}\left[ B \right]\end{eqnarray*}$$

To identify the correct regulatory parts to achieve linear weak positive feedback, we chose monomeric T_7_ RNAP to activate the T_7_ promoter and measured the response curves of six different promoter mutants (with a 150-fold range of varied activities, Supplementary Figure S2B, Supplementary Table S4) by varying T_7_ RNAP expression using a fluorescent reporter gene as an indicator. The experimental data showed that T_7_ RNAP activated the T_7_ promoters with unchanged Hill coefficients of 1.0–1.3 (Figure 1D, Supplementary Figure S2A), indicating that T_7_ RNAP most likely activates the T_7_ promoter in a linear manner; thus, a T_7_ RNAP gene transcribed by a weak T_7_ promoter forms a linear weak positive feedback motif.

We then designed other important regulatory components for the negative and positive regulations between nodes A and B. First, we constructed a hybrid T_7_-cymO promoter and examined how it responded to T_7_ RNAP and Cumate molecule concentrations. As an AND-logic regulatory function, the hybrid promoter was simultaneously activated by T_7_ RNAP and derepressed by CymR repressor by binding to the Cumate molecule. Second, repression from the A-node to the B-node was conducted by a dCpf1-guide RNA (gRNA) complex that contained a gRNA array to target the T_7_ RNAP coding sequences. The superfold gfp (sfgfp) gene was chosen to detect the fluorescent output of the circuit. Combining the T_7_-cymO promoter, dCpf1-gRNA array and the linear weak positive feedback motif together, we created the perfect adaptation circuit (Figure 2A, Supplementary Tables S1 and S2).

Next, we tested the performance of the circuit by switching the external input–Cumate molecule concentration. Despite a nearly 100-fold variation in the concentration of the input molecule and a 20-fold change in the input activity of the P_T7mut+cymO_ promoter, the circuit was able to maintain a relatively constant steady-state output (Figure 2B). When facing step-like switching of input signals, the circuit could generate pulse responses with a similar time scale but different maximal transient expressions according to the input changing folds (Figure 2C, Supplementary Figure S4A). To evaluate the adaptation performance quantitatively, we defined an adaptation error (AErr) value (Eq (3)) to quantify the adaptation performance:

(3) $$\begin{equation*}AErr\ = \frac{{\left| {Outpu{t_1} - Outpu{t_2}} \right|}}{{Outpu{t_1}}}\ \end{equation*}$$

where the variables $Outpu{t_1}$ and $Outpu{t_2}$ are the steady-state output before and after changing the input signal concentrations, respectively. We observed that the AErr values were all small for various Cumate concentrations, indicating that the circuit was robust to perturbations in the input signals. Based on these quantitative measurements, we obtained the parameters by the fitting response curves and transition curves of the individual parts (Figure 2C, Supplementary Figure S3, Supplementary Figure S4A). The predictive ability of the model was evaluated by changing one key parameter - the promoter strength of the B-node. The dynamic response curves of both the stronger and weaker promoters were successfully predicted with the predetermined parameter for promoter activity of the B-node with the same parameter sets as before (Figure 2D, E, Supplementary Figure S4B, C, Supplementary Table S5) (38).

**Figure 3.**
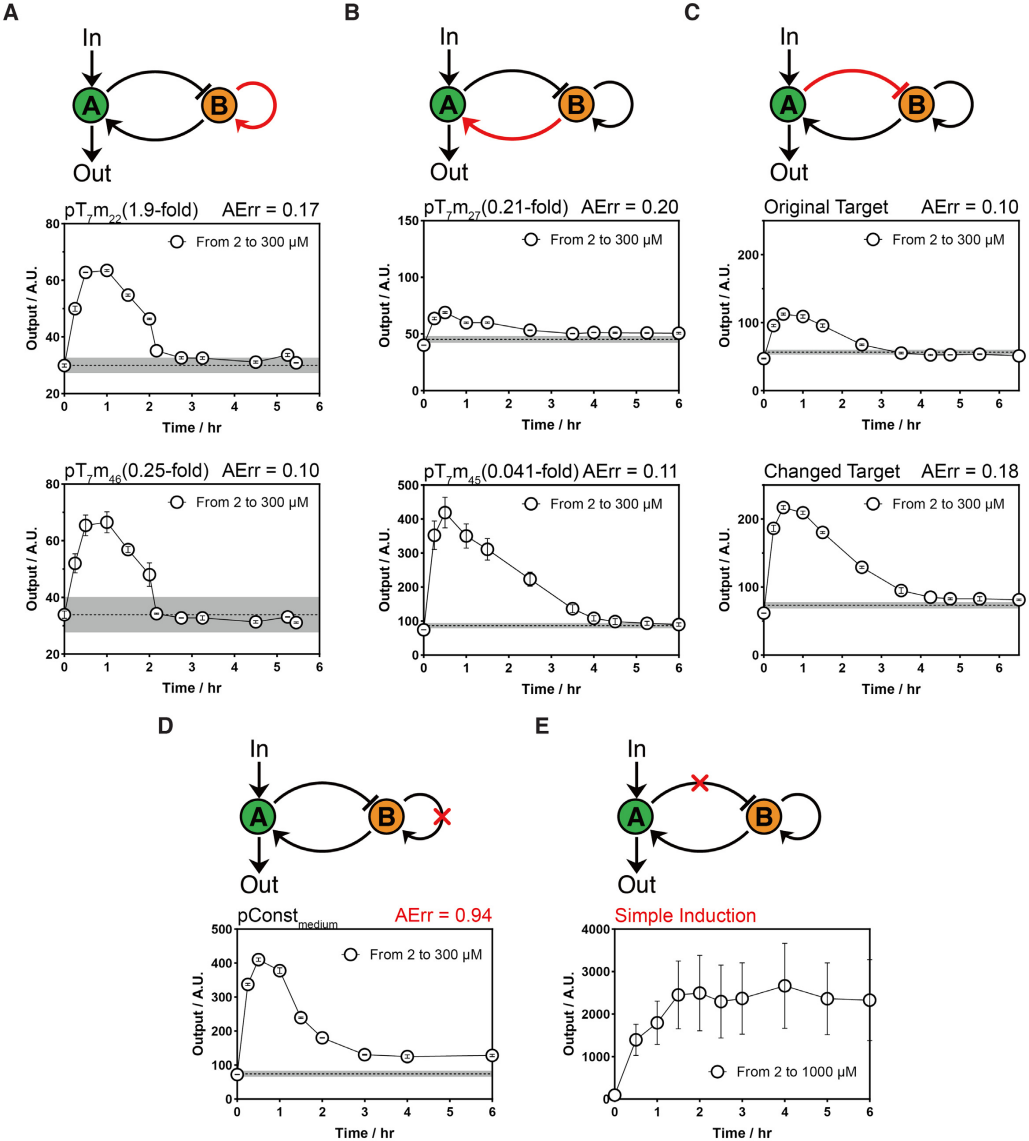
Robustness evaluation of the RPA circuit for single parameter perturbations and topological disruptions. (**A–C**) Schemes and time-course curves of single parameter perturbations for all three types of regulation in the circuit, including autoactivation on node B (A), activation from node B to node A (B) and repression from node A to node B (C). (**D, E**) Schemes and time-course curves for removal of the autoactivation on node B (D) or deletion of the repression from node A to node B (E). Data Information: Time-course curves of each case are shown in the relevant diagrams. For all characterizations, the black points are the mean of the output signal value across all the time series with Cumate concentrations from 2 to 300 μM. The error bar indicates the standard deviation (S.D.) of three independent replicates (*n* = 3). The gray line indicates the mean output signal value across all the time series with a constant Cumate concentration of 2 μM (A–D), and the shade error bar indicates the S.D. of all the recorded data. The perturbation conditions or substitutional genetic parts and the defined AErr index are shown in each subfigure.

According to theoretical analyses, except for the linear property of the buffer node (${n_{BB}} = 1$), perturbation of the other parameters in the circuit topology should not affect the perfect adaptation function (8). We thus individually altered each regulatory interaction within the defined topology to see how it affected the desired adaptation precision. We first perturbed the parameters of the B-node self-activation activity by increasing it up to 2-fold (pT_7_m_22_) or decreasing it 4-fold (pT_7_m_46_) by varying the promoter sequence. The results showed that the adaptation function was still maintained, with both AErr values lower than 0.20 (Figure 3A). Then, we perturbed the node B to node A activation parameter by decreasing the activation strength ∼5-fold (pT_7_m_27_) and 25-fold (pT_7_m_45_, Supplementary Figure S5A). Despite the much stronger perturbation of the activation strength, the final outputs returned to the initial levels with AErr values of 0.20 and 0.11, while the maximal transient expression increased 2-fold and 6-fold relative to the original circuit (Figure 3B). The last regulatory perturbation was the node A to node B repression parameter. The perfect adaptation function was evaluated for the perturbed circuit with different targeted sequences of the dCpf1-gRNA complex repressor (Supplementary Figure S5B). The final expression level of the output recovered to the initial level with different maximal transient and steady-state expression levels (Figure 3C). All the genetic perturbations are summarized in Supplementary Table S3. In conclusion, we systematically changed all the designed interactions in the genetic circuit, and all the tested circuits successfully achieved the defined perfect adaptation function.

Because of the insensitivity to interaction intensities, we next investigated whether it is important to maintain the complete topological structure for the RPA function. We damaged the linear positive feedback on node B by introducing a null positive feedback experiment and a nonlinear positive feedback experiment. To construct null positive feedback controls, three constitutive promoters with different strengths were chosen to replace the T_7_ promoter on the B node (Supplementary Table S3). All these circuits generated imperfect adaptation responses with AErr values of 0.70, 0.94 and 1.3 for these promoters (Figure 3D, Supplementary Figure S7), and a stronger promoter was associated with a larger AErr value. The AErr was slightly decreased when the carbon source switched from glucose to fructose in their growth medium, but their AErr did not perform as well as the original RPA circuit (Supplementary Figure S8). In addition, we utilized nonlinear positive feedback (pR73-φR73δ, with a Hill coefficient of ${n_{BB}} = 2.3$) to replace the original pT_7_-T_7_ RNAP linear positive feedback motif. The bacterial cells were measured by using the mRFP_1_ fluorescent protein marker, and flow cytometry analyses revealed that the bacteria were divided into two populations, indicating that nonlinear positive feedback resulted in more complicated bistable responses before implementing a perfect adaptation function as one homogeneous population (Supplementary Figure S9, Supplementary Table S4). Therefore, null or nonlinear positive feedback on node B resulted in imperfect adaptation, supporting the theoretical implication that linear positive feedback is required for RPA function. To destroy the negative feedback, the repression from node A to node B was removed by deleting the dCpf1 gene (Figure 3E, Supplementary Table S3). As expected, the fluorescence of the output gene dramatically increased without any adapted decrease after switching the Cumate concentration from 2 μM to 1 mM. In conclusion, the experimental results of the incomplete topology indicated that the negative feedback loop is qualitatively dominant in the adaptation, while the results of the parameter perturbation indicated that the linear weak positive feedback plays a quantitative role for adaptation precision and robustness.

**Figure 4.**
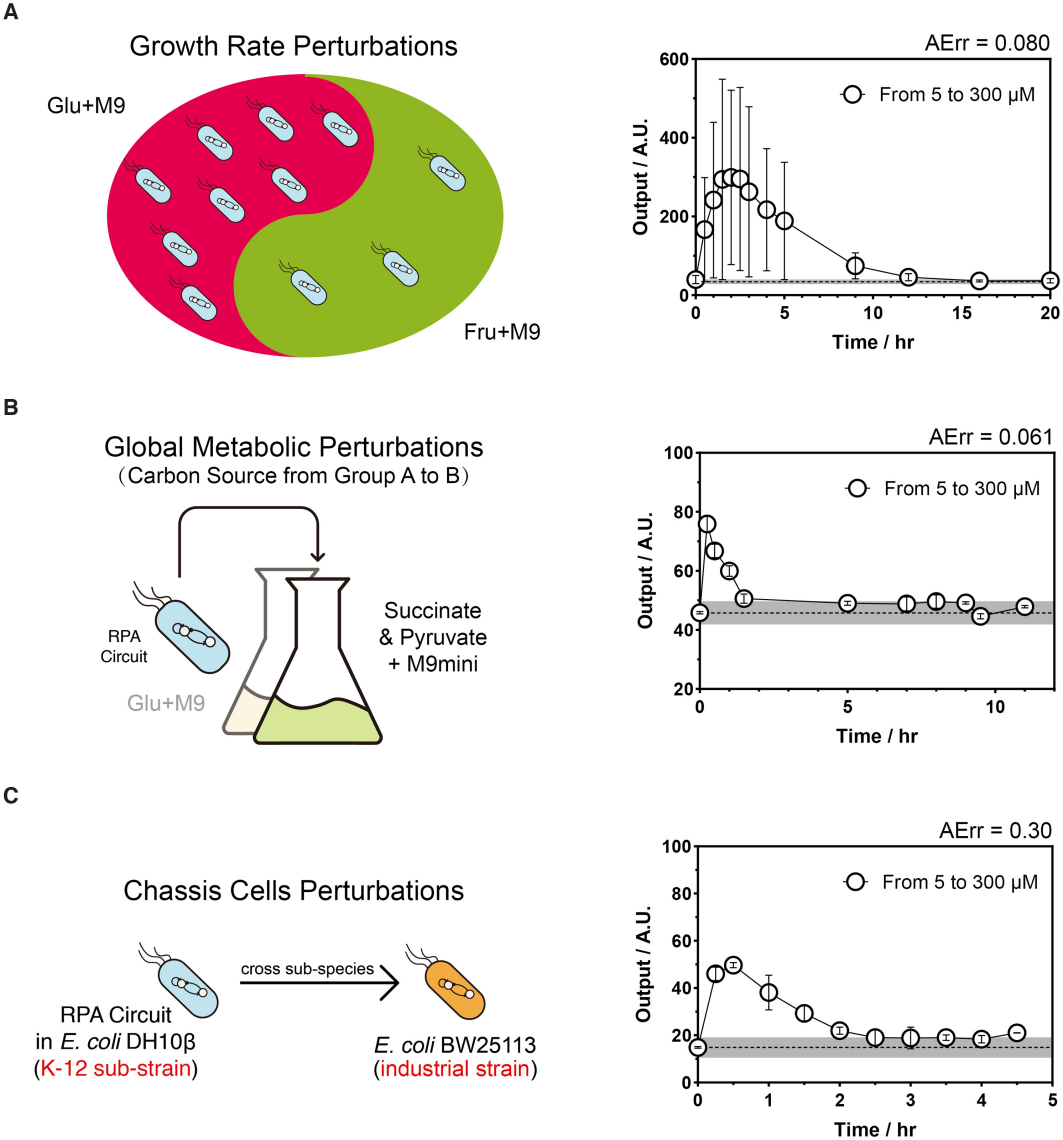
Robustness evaluations of the RPA circuit for global environmental perturbations. (**A–C**) Schemes and time-course curves of global parameter perturbations of the circuit, including changing the growth curve (A), perturbing the metabolic strategy (B) and transferring the circuit into another chassis (C). **Data Information:** Time-course curves for each case are shown in relevant diagrams. For all characterizations, the black points are the mean of the output signal value across all the time series with Cumate concentrations from 5 to 300 μM. The error bar indicates the standard deviation (S.D.) of three independent replicates (*n* = 3). The gray line indicates the mean output signal value across all the time series with a constant Cumate concentration of 5 μM unchanged, and the shade error bar indicates the S.D. of all the recorded data. The defined AErr index is shown in each subfigure.

After elucidating the effects of the single internal interaction in the circuit, we were interested in investigating how well the circuit could maintain its function when facing far more complex environmental fluctuations. First, we changed the carbon source of the growth medium from glucose to fructose. The growth curves of the chassis bacteria in the two media showed significantly different growth rates (Supplementary Figure S6). Although the steady-state level of the output in the fructose medium was changed relative to the glucose medium, the output could also perfectly return to the initial level with an AErr of 0.19 (Figure 4A). Moreover, we evaluated the perfect adaptation function in a complex combined carbon source (succinate and pyruvate). It has been reported that when feeding bacteria carbon sources with different modes of entry into the central metabolic pathway, the bacteria change their carbon flux for more efficient utilization (43,44). Thus, changing the types of carbon sources could perturb the global metabolic strategy and global parameters. We showed that while the instantaneous peak of the response was not as easily observed, the output could still be perfectly restored to the original state, with an AErr of 0.061 (Figure 4B). Finally, encouraged by the previous results, we expected that the circuit with the same topology and same sequence could perform the defined adaptation function in the different strains and species of *E. coli*, even in different genera, e.g. *Pseudomonas*. We first transferred the same RPA circuit from the original chassis bacteria *E. coli* K-12 sub strain DH10B to an industrial strain, BW25113, and the circuit still achieved the defined perfect adaptation function with an AErr of 0.30 (Figure 4C). We further transferred the circuit into another *E. coli* B sub strain, BL21, and crossed genera into an environmentally tolerant strain, *Pseudomonas putida* KT2440, while keeping all the functional sequences and changing the plasmid vector properly. In all conditions, perfect adaptation could be achieved, even though an undetectable pulse appeared within our experimentally temporal solution in some cases (Supplementary Figure S10). In conclusion, all these results indicated that the perfect adaptation function was still well maintained under indirect global parameter perturbations.

## DISCUSSION

Robust perfect adaptation can be found in many natural biological systems, playing key roles in maintaining the response capacity to stimuli and maintaining homeostasis in the presence of internal and external fluctuations (45–47). In this work, we constructed new genetic circuits to achieve perfect adaptation and evaluated their robustness by perturbing their genetic and environmental parameters. Perfect adaptation functions were achieved not only by single-parameter perturbed circuits with single mutated regulatory parts but also by global-parameter perturbed circuits that changed the cell growth rate, carbon sources in the growth medium and even chassis cells. We thus successfully developed a new design strategy for robust genetic circuits by coupling the top-down and bottom-up approaches, and the robust genetic circuit could be transferred from one species or genus to another. As a transient external signal response module, the adaptation circuit could be easily integrated into more sophisticated genetic circuits.

To function as perfect adaptation, several topological genetic circuits were experimentally engineered and estimated their robust capability, including incoherent feedforward loop circuit with the non-cooperative negative regulation (19), integral feedback loop circuit (33) and the negative feedback loop circuit shown here. Based on the differences of their topology, they could buffer the perturbation from different environmental or genetic resources. For example, the incoherent feedforward loop circuit with the non-cooperative negative regulation could stabilize the expression of the output protein in different genome locations and different copy number of the plasmids. The integral feedback loop circuit implemented the perfect adaptation function with a fast time-scale (much faster than the cell growth rate) and exhibited as homeostasis, while our circuit could achieve perfect adaption with a slow time-scale in diverse environmental and genetical conditions.

Synthetic genetic circuits have always shown lower performance than their natural counterparts. Higher-performance synthetic genetic circuits usually require high concentrations of regulatory proteins or synchronization of multiple cells by intercellular signaling systems (48,49), while natural regulatory circuits can utilize very low concentrations of regulatory proteins to achieve similar high-performance regulations. For example, *E. coli* cells only use approximately 10 copies of LacI tetramers to tightly control lactose metabolic enzyme expression (50). The high concentration of synthetic parts would grab too many resources from their host cells and thus induce a growth burden and physiological toxicity to their host (51). The robust design strategy developed here has potential in the building of low-burden and high-performance genetic circuits.

## Supplementary Material

gkac066_Supplemental_File
