# Supplementary material for: Synthetic robust perfect adaptation achieved by negative feedback coupling with linear weak positive feedback
Source: Nucleic Acids Res. 2022 Feb 15;50(4):2377–86. doi: 10.1093/nar/gkac066 (PMC8887471; doi:10.1093/nar/gkac066)
Supplement: gkac066_Supplemental_File [file gkac066_supplemental_file.pdf]

Supplementary Materials

Robust perfect adaptation achieved by negative feedback coupling with linear weak positive feedback

Zhi Sun[1,4], Weijia Wei[2,4], Mingyue Zhang[5,7], Wenjia Shi[8], Yeqing Zong[9], Yihua Chen[2,4], Xiaojing Yang[5,6], Bo Yu[1,4,*], Chao Tang[5,6,7,*] and Chunbo Lou[3,4,*]

[1] CAS Key Laboratory of Microbial Physiological and Metabolic Engineering, State Key Laboratory of Mycology, Institute of Microbiology, Chinese Academy of Sciences, Beijing, 100101, China

[2] State Key Laboratory of Microbial Resources, Institute of Microbiology, Chinese Academy of Sciences, Beijing, 100101, China

[3] Center for Cell and Gene Circuit Design, CAS Key Laboratory of Quantitative Engineering Biology, Guangdong Provincial Key Laboratory of Synthetic Genomics, Shenzhen Key Laboratory of Synthetic Genomics, Shenzhen Institute of Synthetic Biology, Shenzhen Institutes of Advanced Technology, Chinese Academy of Sciences, Shenzhen, 518055, China

[4] College of Life Sciences, University of Chinese Academy of Sciences, Beijing, 100149, China

[5] Center for Quantitative Biology, Academy for Advanced Interdisciplinary Studies, Peking University, Beijing, 100871, China

[6] Peking-Tsinghua Center for Life Sciences, Academy for Advanced Interdisciplinary Studies, Peking University, Beijing, 100871, China

[7] School of Physics, Peking University, Beijing, 100871, China

[8] Department of Applied Physics, School of Sciences, Xi’an University of Technology, Xi’an, 710048, China

[9] Bluepha Co., Ltd, Beijing, 102206, China

Contents

Supplementary Figures 1
- Supplementary Figure 1 1
- Supplementary Figure 2 2
- Supplementary Figure 3 3
- Supplementary Figure 4 4
- Supplementary Figure 5 6
- Supplementary Figure 6 7
- Supplementary Figure 7 8
- Supplementary Figure 8 9
- Supplementary Figure 9 10
- Supplementary Figure 10 11

Details of Plasmid Construction 12
- Supplementary Table 1 12

Details of Parts Substitutions 24
- Supplementary Table 2 24
- Supplementary Table 3 29
- Supplementary Table 4 32

Details of Modeling 34
- Supplementary Table 5 34

Reference 36

Supplementary Figures

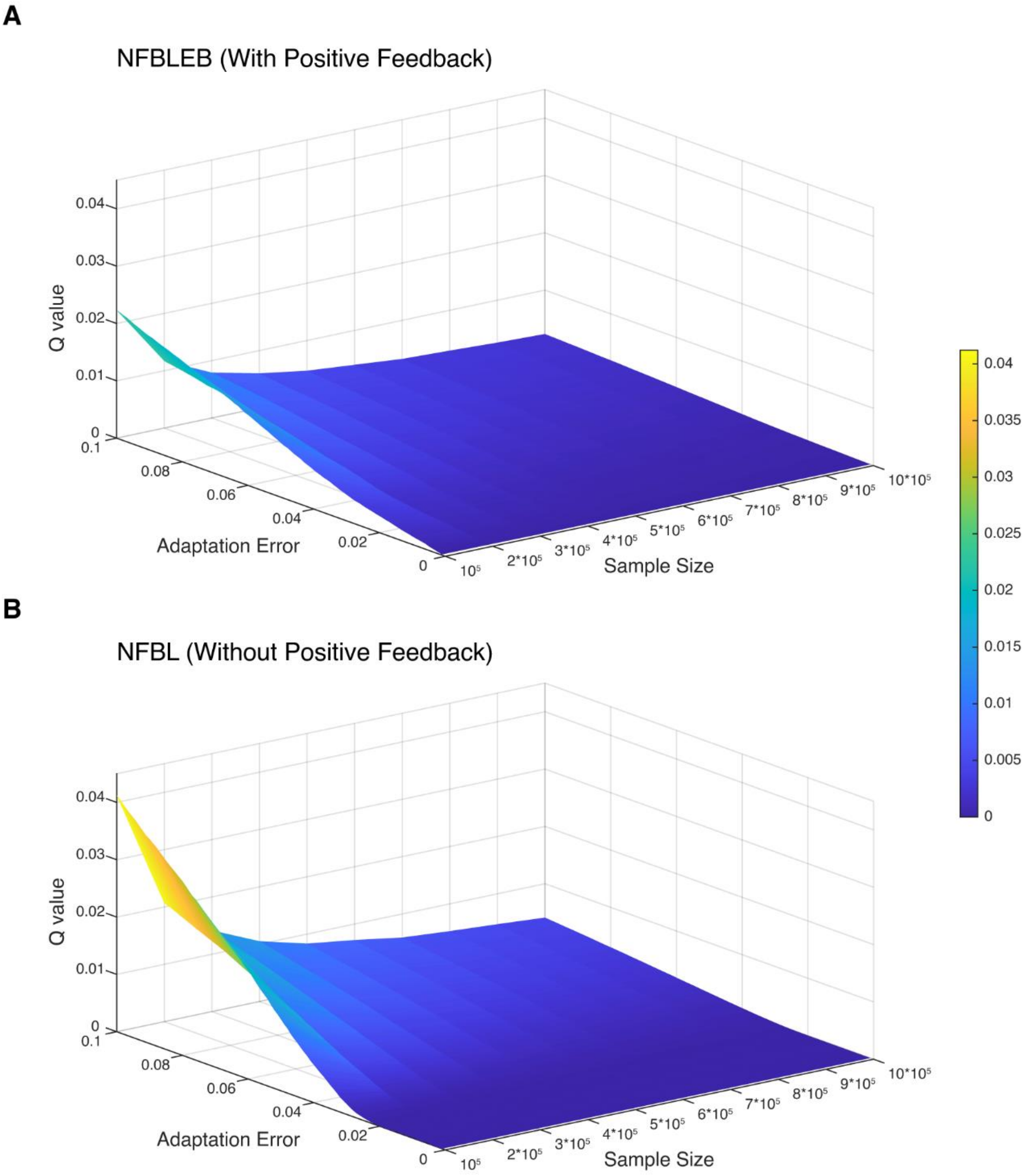

Supplementary Figure 1

The dependences of Q-value on the defined Adaptation Error and sample number in the computational model for the designed RPA circuit with (upper) and without (down) linear positive feedback on B node.

The Q-value is distributed differently depending on the parameters' sampling size and the threshold of Adaptation Error. The parameters are sampled every 100,000 sets, from 100,000 to 1,000,000 sets. The threshold of Adaptation Error is set from 0.001 to 1 with an interval of 0.001.

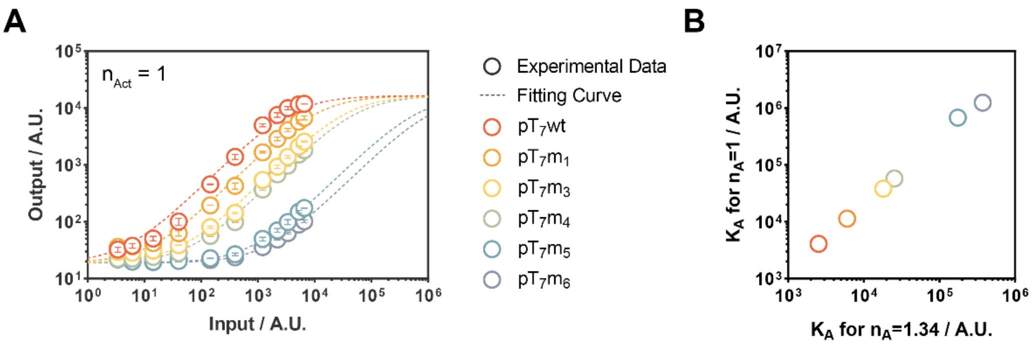

Supplementary Figure 2

Fitting activation curve of T_7 RNAP using different strategies.

A Fitting result of T_7 RNAP activation curve. The Hill Coefficient was limited to 1 (n_{Act}=1). T_7 Promoters with different affinities were indicated in different colors same as Figure 1D in the main text. The dots were the mean values of output signal according to each input values and the error bars indicated standard deviations (S.D.) of three independent replicates (n=3).

B Correlations of promoter affinity between best-fitting (x-axis, according to Figure 1D) and constraint-fitting (y-axis, according to Supplementary Figure 2A). Different promoters were indicated in different colors same as Supplementary Figure 2A.

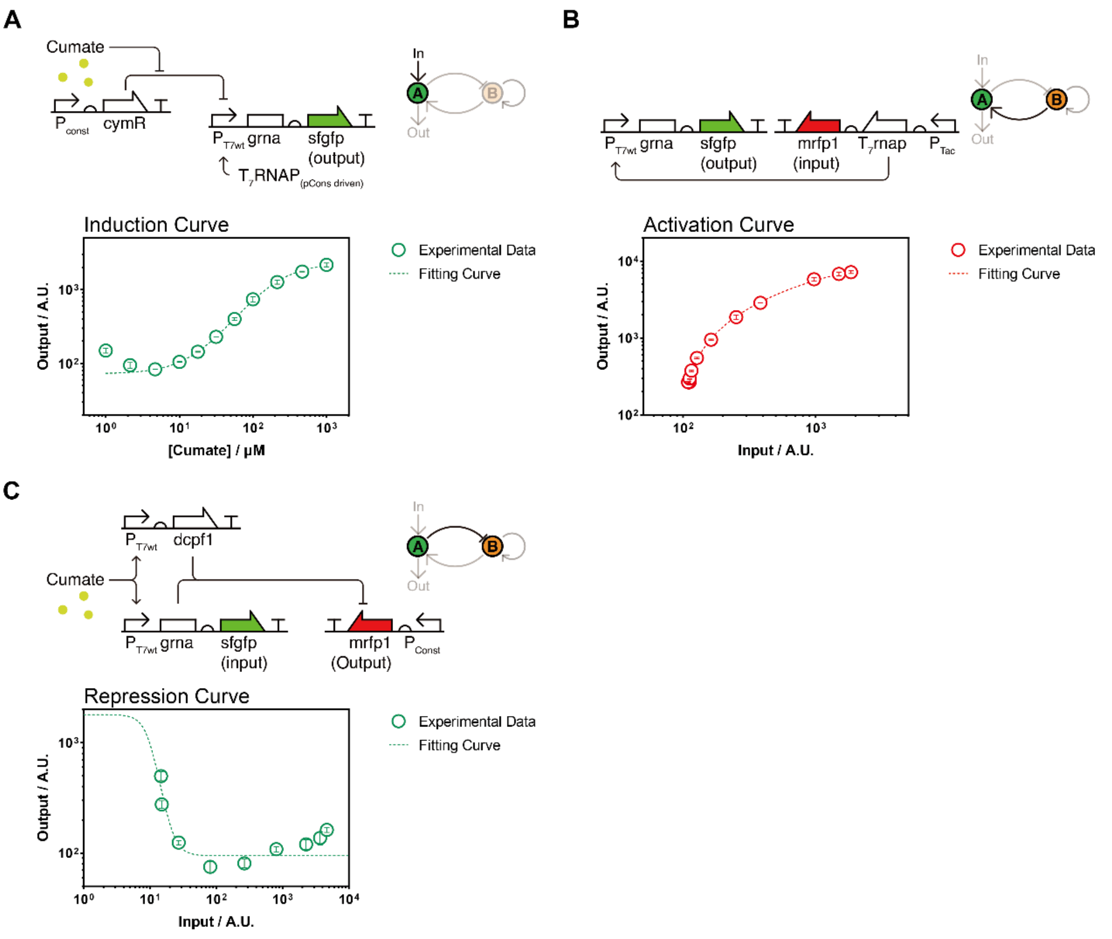

Supplementary Figure 3

Quantitative characterizations for single regulatory parts in the circuit.

A The dose-response curve for the induction regulation from input molecule to A node and reporter gene.

B The input-output curve for the activation regulation from B node to A node.

C The input-output curve for the repression regulation from A node to B node.

Data Information: For each interaction edge, unique systems were constructed (showed above) and measured using inducer concentrations and fluorescent intensity as signals. The blue dots indicate the mean value of experimental data with three biological replicates (n=3). The error bars indicate the standard deviation (S.D.). The red curves are the parameter fitting results.

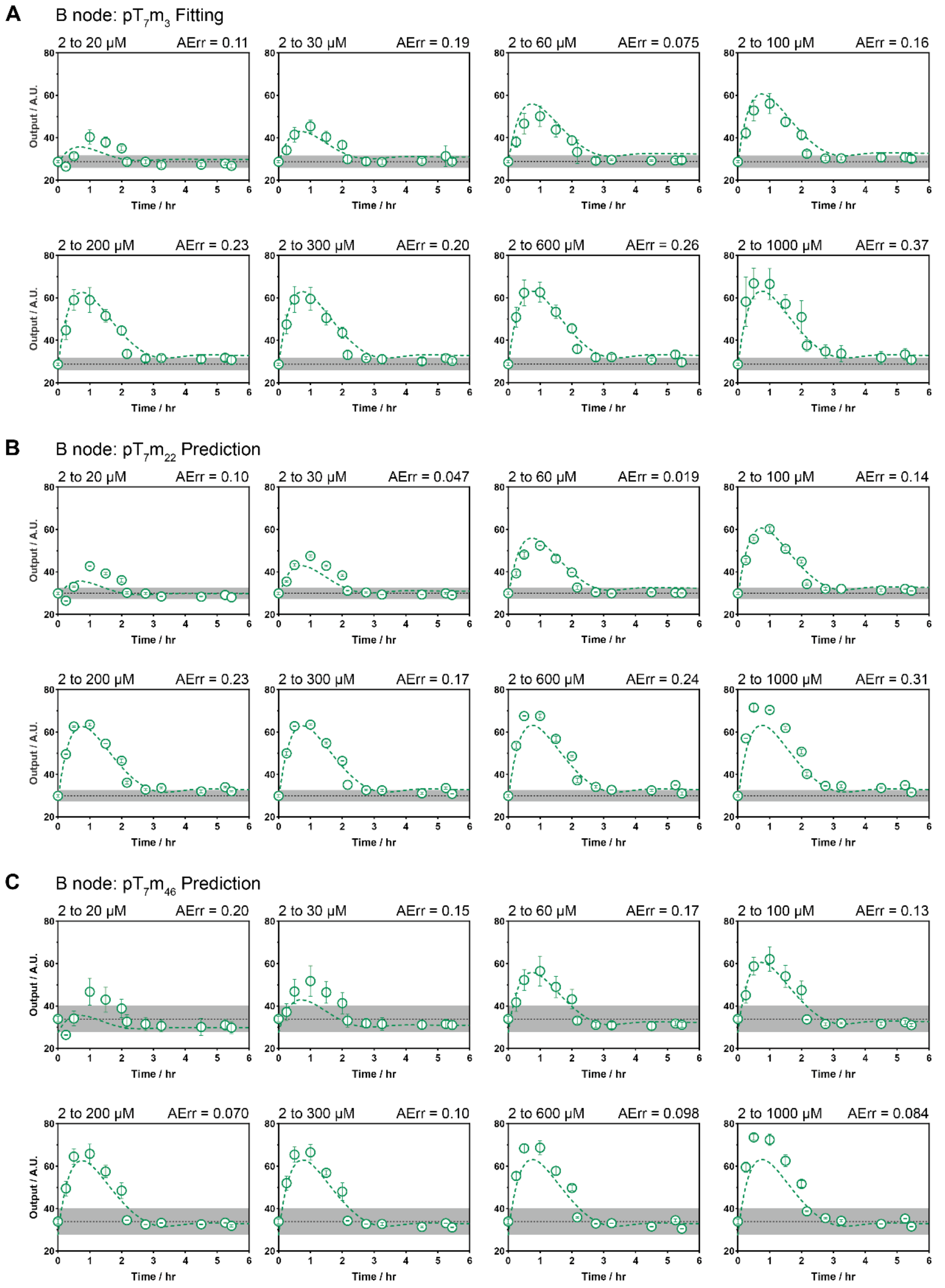

Supplementary Figure 4

Time-course curves of different step-like switching input signals for three different positive feedback activities on the B node.

For all cases, the dots indicate the mean output signal value of experimental data (n = 3) with the Cumate concentration changes from lower to higher level, the error bar indicates the

standard deviation (S.D.). The step-like change happens at Time 0, and the changing input signal level is showed on the top-left for each case. The blue line indicates the mean output signal value cross all the time series with input signal keeps unchanged, and the shade error bar indicates the standard deviation (S.D.). The AErr is showed on the top-right for each case. The pulse-like curves are the simulation for the dynamic behaviors, the curves in (A) are fitting results using pT_7m_3 circuit experimental data and the ones in (B) & (C) are predicting results for pT_7m_{22} and pT_7m_{46} circuits.

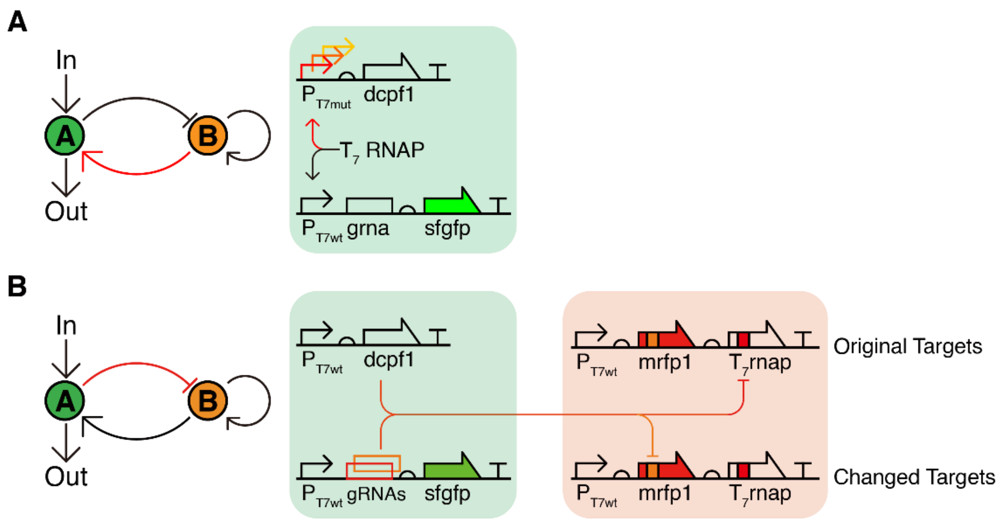

Supplementary Figure 5

The circuit construction details for parameter perturbations.

A Changing the T_7 promoters of dCpf1 to regulate the activation edge from B node to A node.

B Changing the gRNA target sequence and relevant sites to regulate the repression edge from A node to B node. The red rectangle and region indicate the original sequences and target sites and the orange ones indicate the changed ones.

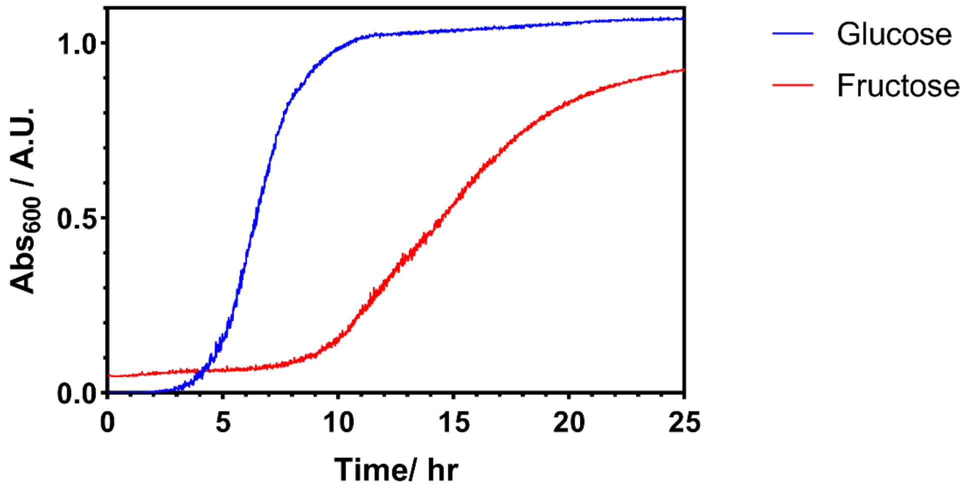

Supplementary Figure 6

The growth curves for the different growth medium of the strains harboring the designed RPA circuit in a turbidostat incubator.

The chassis bacteria grow in minimum M9 medium with either glucose (blue) or fructose (red) as the sole carbon source.

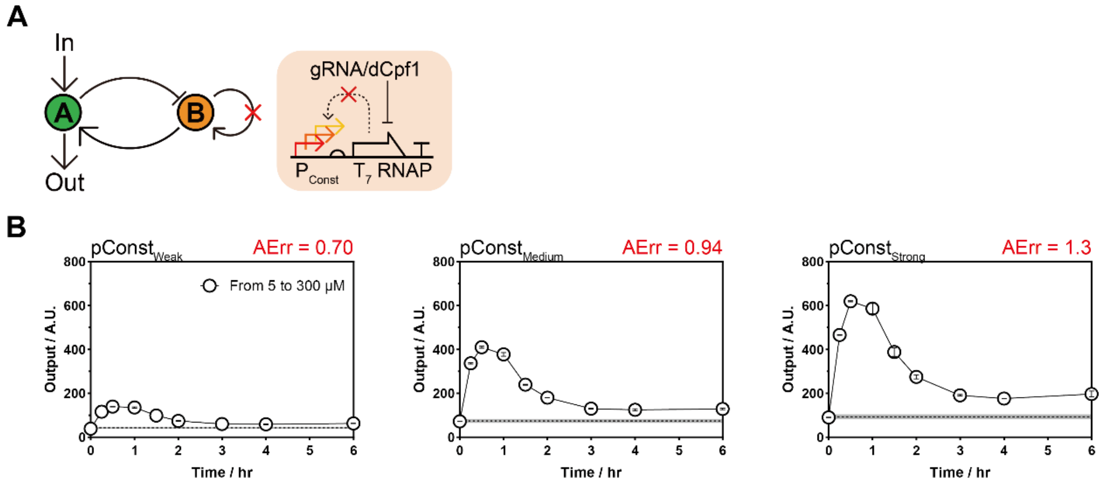

Supplementary Figure 7

The circuit construction details and the time-course curves for the auto-positive-regulation removed circuit.

Several constitutive promoters with different promoter strengths were used to replace the T_7 promoter in B node to damage the self-activation interaction. For all the time-course behavior diagrams, the dots indicate the mean output signal value of experimental data (n = 3) with the Cumate concentration changes from 5 μM to 300 μM, the error bar indicates the standard deviation (S.D.). The gray line indicates the mean output signal value cross all the time series with input signal keeps unchanged, and the shade error bar indicates the standard deviation (S.D.). The substitutional promoters are showed on the top-left and the AErr is showed on the top-right for each case.

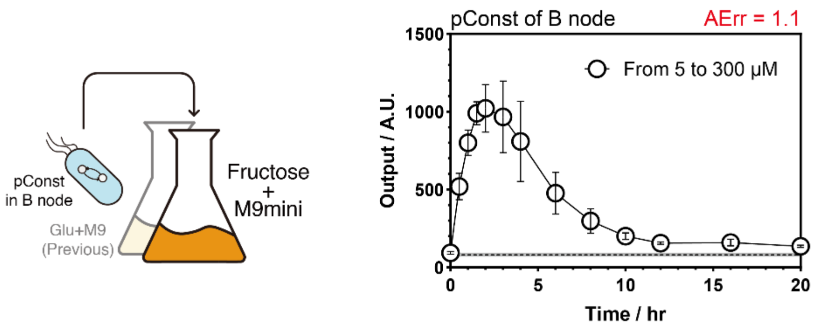

Supplementary Figure 8

The time-course curves for the auto-positive-regulation removed circuit with global parameter perturbations.

The auto-positive-regulation removed circuit with strong promoter strength on the B node was tested in the changed carbon-source condition. The dots indicate the mean output signal value of experimental data (n = 3) with the Cumate concentration changes from 5 μM to 300 μM, the error bar indicates the standard deviation (S.D.). The gray line indicates the mean output signal value cross all the time series with input signal keeps unchanged, and the shade error bar indicates the standard deviation (S.D.). The substitutional promoter is showed on the top-left and the AErr is showed on the top-right.

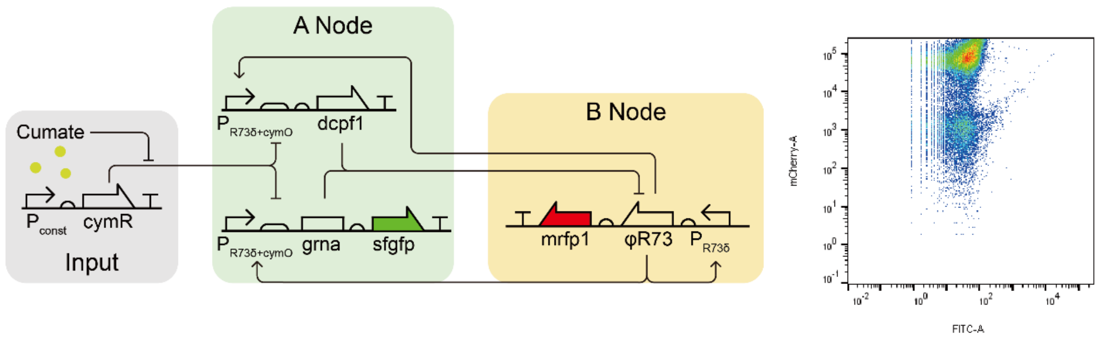

Supplementary Figure 9

Heterogeneous population emerges for the circuit with a higher Hill coefficient auto-positive regulated B node.

Circuit construction details were showed on the left, replacing auto-regulated T_7 RNA Polymerase (Hill Coefficient = 1.0~1.3) with the auto-regulated φR73 activator (Hill Coefficient = 2.3)(1). Relevant promoter was also changed. The flow cytometry data was showed on the right.

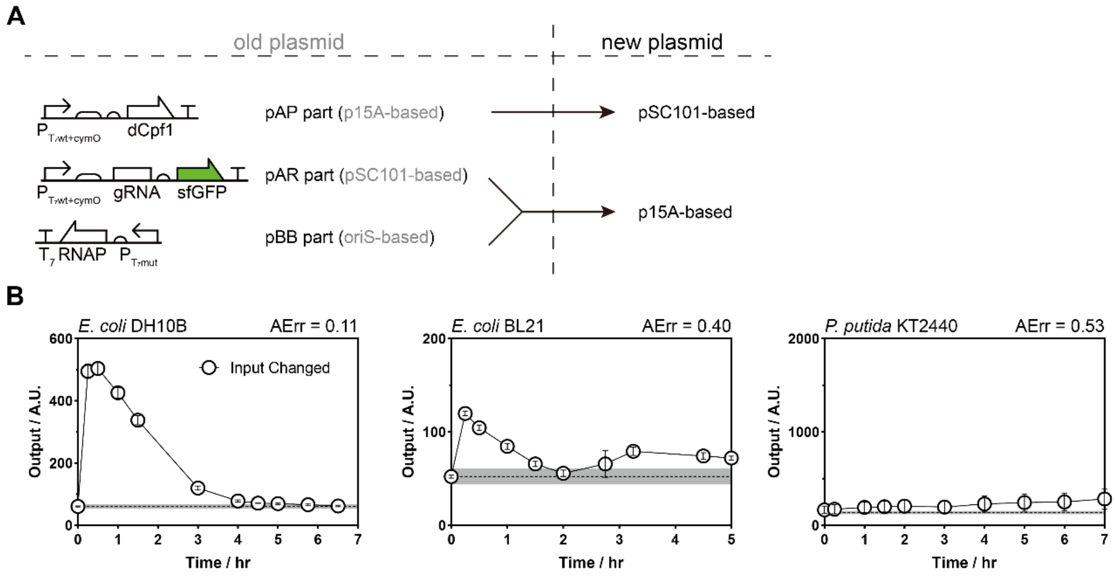

Supplementary Figure 10

The time-course curves for the vector-changed RPA circuit in different chassis cells.

A Graphic illustration of the vector changing. The newly two plasmid backbones were indicated in Supplementary Table 1. The corresponding parts and the assembling strategy were summarized in Supplementary Table 2.

B Time-course curves of the new RPA circuit in different chassis cells, including *E. coli* DH10B (left), *E. coli* BL21 (middle) and *P. putida* KT2440 (right). For all characterizations, the black points are the mean of the output signal value across all the time series with Cumate concentrations changed. The error bar indicates the standard deviation (S.D.) of three independent replicates (n=3). The gray line indicates the mean output signal value across all the time series with the original constant Cumate concentration, and the shade error bar indicates the S.D. of all the recorded data. The defined AErr index are shown in each subplot.

Details of Plasmid Construction

The vast majority of the plasmids used in this study were derived from three basic vectors: pAR, pAP and pBB. The critical parts assembling A node were all constructed on pAR & pAP separately (the gRNA and sfGFP-related cassettes on pAR, the dCpf1-related cassette on pAP) and the ones assembling B node on pBB. For the cross-species test (Supplementary Figure 10), the original three-plasmid system were reconstructed on two plasmids, which include pABn and pAPn.

Supplementary Table 1

Sequence and critical parts on all plasmid backbones.[1]

Plasmid	Function	Sequence (5' - 3')
pAR	Plasmid backbone (Kan^R (in reverse orientation), *pSC101*-based)[2]	tttatactgcaggagtcactaagggttagttagttagattagcagaaagtcaaaagcctccgac<br>cggaggcttttgactaaaacttcccttggggttatcattggggctcactcaaaggcggtaatca<br>gataaaaaaaatccttagctttcgctaaggatgatttctgctagtattaTTAGAAAAACTCATC<br>GAGCATCAAATGAAACTGCAATTTATTCATATCAGGATTATCAATACCATATTTTTGAAAAAGC<br>CGTTTCTGTAATGAAGGAGAAAACTCACCGAGGCAGTTCCAAAGAATGGCAAGGTCCTGGTAAC<br>GGTCTGCGATTCCGACCCGTCCAACATCAATACAACCTATTAATTTCCCCTCGTCAAAAATAAG<br>GTTATCAAGTGAGAAATCACCATGAGTGACGACTGAATCCGGTGAGAATGGCAAGAGCTTGTGC<br>ATTTCTTTCCAGACTTGTTCAACAGGCCAGCCATTACGCTCGTCATCAAAATCACTCGCATCAA<br>CCAAACCGTTATTCATGCGTGATTGCGCCTGAGCAAGACGAAATACACGATCGCTGTTAAAAGG<br>ACAATTACAAACAGGAATCGAATGTAACCGGCGCAGGAACACGGCCAGCGCATCAACAATATTT<br>TCACCTGAATCAGGATATTCTTCTAATACCTGGAAGGCTGTTTTCCCAGGAATCGCGGTGGTGA<br>GTAACCACGCATCATCAGGAGTACGGATAAAATGCTTGATGGTCGGGAGAGGCATAAACTCCGT<br>CAGCCAGTTGAGACGGACCATCTCATCTGTAACATCATTGGCAACGCTACCTTTGCCATGTTTC<br>AGAAACAACTCTGGCGCATCGGGCTTCCCATACAAGCGATAGATTGTCGCACCTGATTGCCCGA<br>CATTATCGCGAGCCCATTTATACCCATATAAATCAGCGTCCATGTTGGAGTTTAAGCGCGGACG<br>GGAGCAAGACGTTTCCCGTTGAATATGGCTCATaacaccccttgtattactgtttatgtaagca<br>gacagttttattgttcatgatgatatatttttatcttgtgcaatgtaacatcagagattttgag<br>acacaacgtggctttgttgaataaatcgaacttttgctgagttgaaggatcagctctagtagtt<br>acattgtcgatctgttcatggtgaacagctttgaatgcaccaaaaactcgtaaaagctctgatg<br>tatctatcttttttacaccgttttcatctgtgcatatggacagttttccctttgatatgtaacg<br>gtgaacagttgttctacttttgtttgttagtcttgatgcttcactgatagatacaagagccata<br>agaacctcagatccttccgtatttagccagtatgttctctagtgtggttcgttgtttttgcgtg<br>agccatgagaacgaaccattgagatcatacttactttgcatgtcactcaaaaattttgcctcaa<br>aactggtgagctgaattttttgcagttaaagcatcgtgtagtgtttttcttagtccgttatgtag<br>gtaggaatctgatgtaatggttgttggtattttgtcaccattcatttttatctggttgttctca<br>agttcggttacgagatccatttgtctatctagttcaacttggaaaatcaacgtatcagtcgggc<br>ggcctcgcttatcaaccaccaatttcatattgctgtaagtgtttaaatctttacttattggttt<br>caaaacccattggttaagccttttaaactcatggtagttattttcaagcattaacatgaactta<br>aattcatcaaggctaatctctatatttgccttgtgagttttcttttgtgttagttcttttaata<br>accactcataaatcctcatagagtatttgttttcaaaagacttaacatgttccagattatattt

		tatgaatttttttaactggaaaagataaggcaatatctcttcactaaaaactaattctaatttt<br>tcgcttgagaacttggcatagtttgtccactggaaaatctcaaagcctttaaccaaaggattcc<br>tgatttccacagttctcgtcatcagctctctggttgctttagctaatacaccataagcattttc<br>cctactgatgttcatcatctgagcgtattggttataagtgaacgataccgtccgttctttcctt<br>gtagggttttcaatcgtggggttgagtagtgccacacagcataaaattagcttggtttcatgct<br>ccgttaagtcatagcgactaatcgctagttcatttgctttgaaaacaactaattcagacataca<br>tctcaattggtctaggtgattttaatcactataccaattgagatgggctagtcaatgataatta<br>catgtccttttcctttgagttgtgggtatctgtaaattctgctagacctttgctggaaaacttg<br>taaattctgctagaccctctgtaaattccgctagacctttgtgtgttttttttgtttatattca<br>agtggttataatttatagaataaagaaagaataaaaaaagataaaaagaatagatcccagccct<br>gtgtataactcactactttagtcagttccgcagtattacaaaaggatgtcgcaaacgctgtttg<br>ctcctctacaaaacagaccttaaaaccctaaaggcttaagtagcaccctcgcaagctcgggcaa<br>atcgctgaatattccttttgtctccgaccatcaggcacctgagtcgctgtctttttcgtgacat<br>tcagttcgctgcgctcacggctctggcagtgaatgggggtaaatggcactacaggcgcctttta<br>tggattcatgcaaggaaactacccataatacaagaaaagcccgtcacgggcttctcagggcgtt<br>ttatggcgggtctgctatgtggtgctatctgacttttttgctgttcagcagttcctgccctctga<br>ttttccagtctgaccacttcggattatcccgtgacaggtcattcagactggctaatgcacccag<br>taaggcagcggtatcatcaacaggcttacccgtcttactgtccctagtgcttggattctcacca<br>ataaaaaacgcccggcggcaaccgagcgttctgaacaaatccagatggagttctgaggtcatta<br>ctggatctatcaacaggagtccaagcgagctcgtaaacttggtctgacagctctagctccggca<br>aaaaaacgggcaaggtgtcaccaccctgcccttttttctttaaaaccgaaaagattacttcgcgt<br>ttgccacctgacgtctaagaaaaggaatattcagcaatttgcccgtgccgaagaaaggcccacc<br>cgtgaaggtgagccagtgagttgattgctacgtaattagttagttagcccttagtgactcgaat<br>tc**tttacggctagctcagtcctaggtatagtgctagctactagagaaagaggagaaatactaga**<br>**tggtaataATGAGCCCAAAAAGAAGAACACAAGCAGAAAGAGCAATGGAAACACAAGGAAAACT**<br>**AATAGCAGCAGCACTAGGAGTACTAAGAGAAAAAGGATACGCAGGATTCAGAATAGCAGACGTA**<br>**CCAGGAGCAGCAGGAGTAAGCAGAGGAGCACAAAGCCACCACTTCCCAACAAAACTAGAACTAC**<br>**TACTAGCAACATTCGAATGGCTATACGAACAAATAACAGAAAGAAGCAGAGCAAGACTAGCAAA**<br>**ACTAAAACCAGAGGACGACGTAATACAACAAATGCTAGACGACGCAGCAGAGTTCTTCCTAGAC**<br>**GACGACTTCAGCATAAGCCTAGACCTAATAGTAGCAGCAGACCGCGACCCAGCACTAAGAGAAG**<br>**GAATACAAAGAACAGTAGAAAGAAACAGATTCGTAGTAGAGGACATGTGGCTAGGAGTGCTAGT**<br>**AAGCAGAGGACTAAGCAGAGACGACGCAGAGGACATACTATGGCTAATATTCAACAGCGTAAGA**<br>**GGACTAGCAGTAAGAAGCCTATGGCAAAAAGACAAAGAAAGATTCGAAAGAGTAAGAAACAGCA**<br>**CACTAGAAATAGCAAGAGAAAGATACGCAAAATTCAAAAGATAAtaactcggtaccaaattcca**<br>**gaaaagaggcctcccgaaagggggcctttttttcgttttggtcc**tactagaggttatgagtcag<br>gaaaaaaggcgacagagtaatctgtcgcctttttttctttgcttgcttttactagag
pAP	Plasmid backbone (Amp^R, *p15A*-based)[3]	tttatactgcaggatttgaacgttgcgaagcaacggcccggagggtggcgggcaggacgcccgc<br>cataaactgccaggcatcaaattaagcagaaggccatcctgacggatggcctttttgcgtttct<br>acaaactcttttgtttatttttctaaatacattcaaatatgtatccgctcatgagacaataacc<br>ctgataaatgcttcaataatattgaaaaaggaagactATGAGTATTCAACATTTCCGTGTCGCC<br>CTTATTCCCTTTTTTGCGGCATTTTGCCTTCCTGTTTTTGCTCACCCAGAAACGCTGGTGAAAG<br>TAAAAGATGCTGAAGATCAGTTGGGTGCACGAGTGGGTTACATCGAACTGGATCTCAACAGCGG<br>TAAGATCCTTGAGAGTTTTCGCCCCGAAGAACGTTTTCCAATGATGAGCACTTTTAAAGTTCTG

		CTATGTGGCGCGGTATTATCCCGTGTTGACGCCGGGCAAGAGCAACTCGGTCGCCGCATACACT<br>ATTCTCAGAATGACTTGGTTGAGTACTCACCAGTCACAGAAAAGCATCTTACGGATGGCATGAC<br>AGTAAGAGAATTATGCAGTGCTGCCATAACCATGAGTGATAACACTGCGGCCAACTTACTTCTG<br>ACAACGATCGGAGGACCGAAGGAGCTAACCGCTTTTTTGCACAACATGGGGGATCATGTAACTC<br>GCCTTGATCGTTGGGAACCGGAGCTGAATGAAGCCATACCAAACGACGAGCGTGACACCACGAT<br>GCCTGCAGCAATGGCAACAACGTTGCGCAAACTATTAACTGGCGAACTACTTACTCTAGCTTCC<br>CGGCAACAATTAATAGACTGGATGGAGGCGGATAAAGTTGCAGGACCACTTCTGCGCTCGGCCC<br>TTCCGGCTGGCTGGTTTATTGCTGATAAATCTGGAGCCGGTGAGCGTGGATCTCGCGGTATCAT<br>TGCAGCACTGGGGCCAGATGGTAAGCCCTCCCGTATCGTAGTTATCTACACGACGGGGAGTCAG<br>GCAACTATGGATGAACGAAATAGACAGATCGCTGAGATAGGTGCCTCACTGATTAAGCATTGGT<br>AActgtcagaccaagtttactcatatatactttagattgatttacgcgccctgtagcggcgcat<br>taagcgcggcgggtgtggtggttacgcgcagcgtgaccgctacacttgccagcgccctagcgcc<br>cgctcctttcgctttcttcccttcctttctcgccacgttcgccggctttccccgtcaagctcta<br>aatcgggggctccctttagggttccgatttagtgctttacggcacctcgaccccaaaaaacttg<br>atttgggtgatggttcacgtagtgggccatcgccctgatagacggtttttcgccctttgacgtt<br>ggagtccacgttctttaatagtggactcttgttccaaacttgaacaacactcaaccctatctcg<br>ggctattcttttgatttataagggattttgccgatttcggcctattggttaaaaaatgagctga<br>tttaacaaaaatttaacgcgaattttaacaaaatattaacgtttacaatttaaaaggatctagg<br>tgaagatcctttttgataatctcatgaccaaaatcccttaacgtgagttttcgttccactgagc<br>gtcagaccccgtagaaaagatcaaaggatcttcttgagatcctttttttctgcgcgtaatctgc<br>tgcttgcaaacaaaaaaaccaccgctaccagcggtggtttgtttgccggatcaagagctaccaa<br>ctctttttccgaaggtaactggcttcagcagagcgcagataccaaatactgtccttctagtgta<br>gccgtagttaggccaccacttcaagaactctgtagcaccgcctacatacctcgctctgctaatc<br>ctgttaccagtcaggcatttgagaagcacacggtcacactgcttccggtagtcaataaaccggt<br>aaaccagcaatagacataagcggctatttaacgaccctgccctgaaccgacgaccgggtcgaat<br>ttgctttcgaatttctgccattcatccgcttattatcacttattcaggcgtagcaccaggcgtt<br>taagggcaccaataactgccttaaaaaaattacgccccgccctgccactcatcgcagtactgtt<br>gtaattcattaagcattctgccgacatggaagccatcacagacggcatgatgaacctgaatcgc<br>cagcggcatcagcaccttgtcgccttgcgtataatatttgcccatggctagcggagtgtatact<br>ggcttactatgttggcactgatgagggtgtcagtgaagtgcttcatgtggcaggagaaaaaagg<br>ctgcaccggtgcgtcagcagaatatgtgatacaggatatattccgcttcctcgctcactgactc<br>gctacgctcggtcgttcgactgcggcgagcggaaatggcttacgaacggggcggagatttcctg<br>gaagatgccaggaagatacttaacagggaagtgagagggccgcggcaaagccgtttttccatag<br>gctccgcccccctgacaagcatcacgaaatctgacgctcaaatcagtggtggcgaaacccgaca<br>ggactataaagataccaggcgtttccccctggcggctccctcgtgcgctctcctgttcctgcct<br>ttcggtttaccggtgtcattccgctgttatggccgcgtttgtctcattccacgcctgacactca<br>gttccgggtaggcagttcgctccaagctggactgtatgcacgaaccccccgttcagtccgaccg<br>ctgcgccttatccggtaactatcgtcttgagtccaacccggaaagacatgcaaaagcaccactg<br>gcagcagccactggtaattgatttagaggagttagtcttgaagtcatgcgccggttaaggctaa<br>actgaaaggacaagttttggtgactgcgctcctccaagccagttacctcggttcaaagagttgg<br>tagctcagagaaccttcgaaaaaccgccctgcaaggcggttttttcgttttcagagcaagagat<br>tacgcgcagaccaaaacgatctcaagaagatcatcttattaatcagataaaatatttgctcatg<br>agcccgaagtggcgagcccgatcttccccatcggtgatgtcggcgatataggcgccagcaaccg

		cacctgtggcgccggtgatgccggccacgatgcgtccggcgtagaggatctgctcatgtttgac<br>agcttatcatcgatgcataatgtgcctgtcaaatggacgaagcagggattctgcaaaccctatg<br>ctactccctcgagccgtcaattgtctgattcgttaccaattagaattc**TCACTGCCCGCTTTCC**<br>**AGTCGGGAAACCTGTCGTGCCAGCTGCATTAATGAATCGGCCAACGCGCGGGGAGAGGCGGTTT**<br>**GCGTATTGGGCGCCAGGGTGGTTTTTCTTTTCACCAGTGAGACTGGCAACAGCTGATTGCCCTT**<br>**CACCGCCTGGCCCTGAGAGAGTTGCAGCAAGCGGTCCACGCTGGTTTGCCCCAGCAGGCGAAAA**<br>**TCCTGTTTGATGGTGGTTAACGGCGGGATATAACATGAGCTATCTTCGGTATCGTCGTATCCCA**<br>**CTACCGAGATATCCGCACCAACGCGCAGCCCGGACTCGGTAATGGCGCGCATTGCGCCCAGCGC**<br>**CATCTGATCGTTGGCAACCAGCATCGCAGTGGGAACGATGCCCTCATTCAGCATTTGCATGGTT**<br>**TGTTGAAAACCGGACATGGCACTCCAGTCGCCTTCCCGTTCCGCTATCGGCTGAATTTGATTGC**<br>**GAGTGAGATATTTATGCCAGCCAGCCAGACGCAGACGCGCCGAGACAGAACTTAATGGGCCCGC**<br>**TAACAGCGCGATTTGCTGGTGACCCAATGCGACCAGATGCTCCACGCCCAGTCGCGTACCGTCC**<br>**TCATGGGAGAAAATAATACTGTTGATGGGTGTCTGGTCAGAGACATCAAGAAATAACGCCGGAA**<br>**CATTAGTGCAGGCAGCTTCCACAGCAATGGCATCCTGGTCATCCAGCGGATAGTTAATGATCAG**<br>**CCCACTGACGCGTTGCGCGAGAAGATTGTGCACCGCCGCTTTACAGGCTTCGACGCCGCTTCGT**<br>**TCTACCATCGACACCACCACGCTGGCACCCAGTTGATCGGCGCGAGATTTAATCGCCGCGACAA**<br>**TTTGCGACGGCGCGTGCAGGGCCAGACTGGAGGTGGCAACGCCAATCAGCAACGACTGTTTGCC**<br>**CGCCAGTTGTTGTGCCACGCGGTTGGGAATGTAATTCAGCTCCGCCATCGCCGCTTCCACTTTT**<br>**TCCCGCGTTTTCGCAGAAACGTGGCTGGCCTGGTTCACCACGCGGGAAACGGTCTGATAAGAGA**<br>**CACCGGCATACTCTGCGACATCGTATAACGTTACTGGTTTCAT**attcaccaccctgaattgact<br>ctcttccgggcgctatcatgccataccgcgaaaggttttgcgccattcgatggcgcgccgcttc<br>gtcaggccacatagctttcttgttctgatcggaacgatcgttggctgctcgagccgtcaattgt<br>ctgattcgttaccaattagaattcctcggtaccaaattccagaaaagaggcctcccgaaagggg<br>ggcctttttttcgttttggtcctactagaggttatgagtcaggaaaaaaggcgacagagtaatct<br>gtcgcctttttttctttgcttgcttttactagag
pBB	Plasmid backbone (Apr^R, *oriS*-based)	tactagagctgcagcactcgttgctttatcggtatattaccctgttatccctaattttgttatc<br>aataaaaaaggcccccgttagggaggccttattgttcgtcgctcactcaaaggcggtaatgac<br>gctcagtggaacgaaaactcacgttaagggtaaaactgtattataagtaaatgcatgtatacta<br>aactcacaaattagagcttcaatttaattatatcagttattacccataacttcgtatagcatac<br>attatacgaagttatcccgggtaccgagctcgattcgtaacttacacgcgcctcgtatcttta<br>atgatggaataatttgggaatttactctgtgtttatttatttttatgttttgtatttggatttt<br>agaaagtaaataaagaaggtagaagagttacggaatgaagaaaaaaaaataaacaaaggtttaa<br>aaaatttcaacaaaaagcgtactttacatatatatttattagacaagaaaagcagattaaatag<br>atatacattcgattaacgataagtaaaatgtaaaatcacaggattttcgtgtgtggtcttctac<br>acagacaagatgaaacaattcggcattaatacctgagagcaggaagagcaagataaaaggtagt<br>atttgttggcgatccccctagagtcttttacatcttcggaaaacaaaaactatttttttctttaa<br>tttcttttttttactttctatttttaatttatatatttatattaaaaaatttaaattataattat<br>ttttatagcacgtgagatcaacgtctcattttcgccaaaagttggcccagggcttcccggtatc<br>aacagggacaccaggatttatttattctgcgaagtgatcttccgtcacaggtatttattcgcga<br>taagctcatggagcggcgtaaccgtcgcacaggaaggacagagaaagcgcggatctgggaagtg<br>acggacagaacggtcaggacctggattggggaggcggttgccgccgctgctgctgacggtgtga<br>cgttctctgttccggtcacaccacatacgttccgccattcctatgcgatgcacatgctgtatgc<br>cggtataccgctgaaagttctgcaaagcctgatgggacataagtccatcagttcaacggaagtc

		tacacgaaggtttttgcgctggatgtggctgcccggcaccgggtgcagtttgcgatgccggagt<br>ctgatgcggttgcgatgctgaaacaattatcctgagaataaatgccttggcctttatatggaaa<br>tgtggaactgagtggatatgctgtttttgtctgttaaacagagaagctggctgttatccactga<br>gaagcgaacgaaacagtcgggaaaatctcccattatcgtagagatccgcattattaatctcagg<br>agcctgtgtagcgtttataggaagtagtgttctgtcatgatgcctgcaagcggtaacgaaaacg<br>atttgaatatgccttcaggaacaatagaaatcttcgtgcggtgttacgttgaagtggagcggat<br>tatgtcagcaatggacagaacaacctaatgaacacagaaccatgatgtggtctgtccttttaca<br>gccagtagtgctcgccgcagtcgagcgacagggcgaagccctcgagctggttgccctcgccgct<br>gggctggcggccgtctatggccctgcaaacgcgccagaaacgccgtcgaagccgtgtgcgagac<br>accgcggccggccgccggcgttgtggatacctcgcggaaaacttggccctcactgacagatgag<br>gggcggacgttgacacttgaggggccgactcacccggcgcggcgttgacagatgaggggcaggc<br>tcgatttcggccggcgacgtggagctggccagcctcgcaaatcggcgaaaacgcctgattttac<br>gcgagtttcccacagatgatgtggacaagcctggggataagtgccctgcggtattgacacttga<br>ggggcgcgactactgacagatgaggggcgcgatccttgacacttgaggggcagagtgctgacag<br>atgaggggcgcacctattgacatttgaggggctgtccacaggcagaaaatccagcatttgcaag<br>ggtttccgcccgtttttcggccaccgctaacctgtcttttaacctgcttttaaaccaatattta<br>taaaccttgtttttaaccagggctgcgccctgtgcgcgtgaccgcgcacgccgaagggggggtgc<br>ccccccttctcgaaccctcccggtcgagtgagcgaggaagcaccagggaacagcacttatatat<br>tctgcttacacacgatgcctgaaaaaacttcccttggggttatccacttatccacggggatatt<br>tttataattattttttttatagtttttagatcttctttttttagagcgccttgtaggcctttatc<br>catgctggttcatgagaaggtgttgtgacaaattgccctttcagtgtgacaaatcaccctcaaa<br>tgacagtcctgtctgtgacaaattgcccttaaccctgtgacaaattgccctcagaagaagctgt<br>tttttcacaaagttatccctgcttattgactctttttttatttagtgtgacaatctaaaaacttg<br>tcacacttcacatggatctgtcatggcggaaacagcggttatcaatcacaagaaacgtaaaaat<br>agcccgcgaatcgtccagtcaaacgacctcactgaggcggcatatagtctctcccgggatcaaa<br>aacgtatgctgtatctgttcgttgaccagatcagaaaatctgatggcaccctacaggaacatga<br>cggtatctgcgagatccatgttgctaaatatgctgaaatattcggattgacctctgcggaagcc<br>agtaaggatatacggcaggcattgaagagtttcgcggggaaggaagtggtttttttatcgccctg<br>aagaggatgccggcgatgaaaaaggctatgaatcttttccttggtttatcaaacgtgcgcacag<br>tccatccagagggctttacagtgtacatatcaacccatatctcattcccttctttatcgggtta<br>cagaaccggtttacgcagtttcggcttagtgaaacaaaagaaatcaccaatccgtatgccatgc<br>gtttatacgaatccctgtgtcagtatcgtaagccggatggctcaggcatcgtctctctgaaaat<br>cgactggatcatagagcgttaccagctgcctcaaagttaccagcgtatgcctgacttccgccgc<br>cgcttcctccaggtctgtgttaatgagatcaacagcagaactccaatgcgcctctcatacattg<br>agaaaaagaaaggccgccagacgactcatatcgtattttccttccgcgatatcacttccatgac<br>gacaggatagtctgagggttatctgtcacagatttgagggtggttcgtcacatttgttctgacc<br>tactgagggtaatttgtcacagttttgctgtttccttcagcctgcatggattttctcatacttt<br>ttgaactgtaattttttaaggaagccaaatttgagggcagtttgtcacagttgatttccttctct<br>ttcccttcgtcatgtgacctgatatcgggggttagttcgtcatcattgatgagggttgattatc<br>acagtttattactctgaattggctatccgcgtgtgtacctctacctggagtttttcccacggtg<br>gatatttcttcttgcgctgagcgtaagagctatctgacagaacagttcttctttgcttcctcgc<br>cagttcgctcgctatgctcggttacacggctgcggcgagcgctagtgataataagtgactgagg<br>tatgtgctcttcttatctccttttgtagtgttgctcttattttaaacaactttgcggtttttttg

		atgactttgcgattttgttgttgctttgcagtaaattgcaagatttaataaaaaaacgcaaagc<br>aatgattaaaggatgttcagaatgaaactcatggaaacacttaaccagtgcataaacgctggtc<br>atgaaatgacgaaggctatcgccattgcacagtttaatgatgacagcccggaagcgaggaaaat<br>aacccggcgctggagaataggtgaagcagcggatttagttggggtttcttctcaggctatcaga<br>gatgccgagaaagcagggcgactaccgcacccggatatggaaattcgaggacgggttgagcaac<br>gtgttggttatacaattgaacaaattaatcatatgcgtgatgtgtttggtacgcgattgcgacg<br>tgctgaagacgtatttccaccggtgatcggggttgctgcccataaaggtggcgtttacaaaacc<br>tcagtttctgttcatcttgctcaggatctggctctgaaggggctacgtgttttgctcgtggaag<br>gtaacgacccccagggaacagcctcaatgtatcacggatgggtaccagatcttcatattcatgc<br>agaagacactctcctgcctttctatcttggggaaaaggacgatgtcacttatgcaataaagccc<br>acttgctggccggggcttgacattattccttcctgtctggctctgcaccgtattgaaactgagt<br>taatgggcaaatttgatgaaggtaaactgcccaccgatccacacctgatgctccgactggccat<br>tgaaactgttgctcatgactatgatgtcatagttattgacagcgcgcctaacctgggtatcggc<br>acgattaatgtcgtatgtgctgctgatgtgctgattgttcccacgcctgctgagttgtttgact<br>acacctccgcactccagtttttcgatatgcttcgtgatctgctcaagaacgttgatcttaaagg<br>gttcgagcctgatgtacgtattttgcttaccaaatacagcaatagtaatggctctcagtccccg<br>tggatggaggagcaaattcgggatgcctggggaagcatggttctaaaaaatgttgtacgtgaaa<br>cggatgaagttggtaaaggtcagatccggatgagaactgtttttgaacaggccattgatcaacg<br>ctcttcaactggtgcctggagaaatgctctttctatttgggaacctgtctgcaatgaaattttc<br>gatcgtctgattaaaccacgctgggagattagataatgaagcgtgcgcctgttattccaaaaca<br>tacgctcaatactcaaccggttgaagatacttcgttatcgacaccagctgccccgatggtggat<br>tcgttaattgcgcgcgtaggagtaatggctcgcggtaatgccattactttgcctgtatgtggtc<br>gggatgtgaagtttactcttgaagtgctccggggtgatagtgttgagaagacctctcgggtatg<br>gtcaggtaatgaacgtgaccaggagctgcttactgaggacgcactggatgatctcatcccttct<br>tttctactgactggtcaacagacaccggcgttcggtcgaagagtatctggtgtcatagaaattg<br>ccgatgggagtcgccgtcgtaaagctgctgcacttaccgaaagtgattatcgtgttctggttgg<br>cgagctggatgatgagcagatggctgcattatccagattgggtaacgattatcgcccaacaagt<br>gcttatgaacgtggtcagcgttatgcaagccgattgcagaatgaatttgctggaaatatttctg<br>cgctggctgatgcggaaaatatttcacgtaagattattacccgctgtatcaacaccgccaaatt<br>gcctaaatcagttgttgctcttttttctcaccccggtgaactatctgcccggtcaggtgatgca<br>cttcaaaaagcctttacagataaagaggaattacttaagcagcaggcatctaaccttcatgagc<br>agaaaaaagctggggtgatatttgaagctgaagaagttatcactcttttaacttctgtgcttaa<br>aacgtcatctgcatcaagaacaagtttaagctcacgacatcagtttgctcctggagcgacagta<br>ttgtataagggcgataaaatggtgcttaacctggacaggtcccgtgttccaactgagtgtatag<br>agaaaattgaggccattcttaaggaacttgaaaagccagcaccctgatgcgaccacgttttagt<br>ctacgtttatctgtctttacttaatgtcctttgttacaggccagaaagcataactggcctgaat<br>attctctctgggcccactgttccacttgtatcgtcggtctgataatcagactgggaccacggtc<br>ccactcgtatcgtcggtctgattattagtctgggaccacggtcccactcgtatcgtcggtctga<br>ttattagtctgggaccacggtcccactcgtatcgtcggtctgataatcagactgggaccacggt<br>cccactcgtatcgtcggtctgattattagtctgggaccatggtcccactcgtatcgtcggtctg<br>attattagtctgggaccacggtcccactcgtatcgtcggtctgattattagtctggaaccacgg<br>tcccactcgtatcgtcggtctgattattagtctgggaccacggtcccactcgtatcgtcggtct<br>gattattagtctgggaccacgatcccactcgtgttgtcggtctgattatcggtctgggaccacg

		gtcccacttgtattgtcgatcagactatcagcgtgagactacgattccatcaatgcctgtcaag<br>ggcaagtattgacatgtcgtcgtaacctgtagaacggagtaacctcggtgtgcggttgtatgcc<br>tgctgtggattgctgctgtgtcctgcttatccacaacattttgcgcacggttatgtggacaaaa<br>tacctgattttggtcatgagattatcaaaaaggatcttcacctagatccttttggttcatgtgc<br>agctccatcagcaaaaggggatgataagtttatcaccaccgactatttgcaacagtgccgttga<br>tcgtgctatgatcgactgATGTCATCAGCGGTGGAGTGCAATGTCGTGCAATACGAATGGCGAA<br>AAGCCGAGCTCATCGGTCAGCTTCTCAACCTTGGGGTTACCCCCGGCGGTGTGCTGCTGGTCCA<br>CAGCTCCTTCCGTAGCGTCCGGCCCCTCGAAGATGGGCCACTTGGACTGATCGAGGCCCTGCGT<br>GCTGCGCTGGGTCCGGGAGGGACGCTCGTCATGCCCTCGTGGTCAGGTCTGGACGACGAGCCGT<br>TCGATCCTGCCACGTCGCCCGTTACACCGGACCTTGGAGTTGTCTCTGACACATTCTGGCGCCT<br>GCCAAATGTAAAGCGCAGCGCCCATCCATTTGCCTTTGCGGCAGCGGGGCCACAGGCAGAGCAG<br>ATCATCTCTGATCCATTGCCCCTGCCACCTCACTCGCCTGCAAGCCCGGTCGCCCGTGTCCATG<br>AACTCGATGGGCAGGTACTTCTCCTCGGCGTGGGACACGATGCCAACACGACGCTGCATCTTGC<br>CGAGTTGATGGCAAAGGTTCCCTATGGGGTGCCGAGACACTGCACCATTCTTCAGGATGGCAAG<br>TTGGTACGCGTCGATTATCTCGAGAATGACCACTGCTGTGAGCGCTTTGCCTTGGCGGACAGGT<br>GGCTCAAGGAGAAGAGCCTTCAGAAGGAAGGTCCAGTCGGTCATGCCTTTGCTCGGTTGATCCG<br>CTCCCGCGACATTGTGGCGACAGCCCTGGGTCAACTGGGCCGAGATCCGTTGATCTTCCTGCAT<br>CCGCCAGAGGCGGGATGCGAAGAATGCGATGCCGCTCGCCAGTCGATTGGCTGAgctcatgagc<br>ggagaacgagatgacgttggaggggcaaggtcgcgctgattgctggggcaacacgtggagcgga<br>tcggggattgtctttcttcagctcgctgatgatatgctgacgctcaatgccgtttggcctccga<br>ctaacgaaaatcccgcatttggacggctgatccgattggcacggcggacggcgaatggcggagc<br>agacgctcgtccgggggcaatgagatatgaaaaagcctgaactcaccgcgacgtatcgggccct<br>ggccagctagctagagtcgacctgggtccccggggatcggtcttgccttgctcgtcggtgatgt<br>acttcaccagctccgcgaagtcgctcttcttgatggagcgcatggggacgtgcttggcaatcac<br>gcgcacccccggccgttttagcggctaaaaaagtcatggctctgccctcgggcggaccacgcc<br>catcatgaccttgccaagctcgtcctgcttctcttcgatcttcgccagcagggcgaggatcgtg<br>gcatcaccgaaccgcgccgtgcgcgggtcgtcggtgagccagagtttcagcaggccgcccaggc<br>ggcccaggtcgccattgatgcgggccagctcgcggacgtgctcatagtccacgacgcccgtgat<br>tttgtagccctggccgacggccagcaggtaggccgacaggctcatgccggccgccgccgccttt<br>tcctcaatcgctcttcgttcgtctggaaggcagtacaccttgataggtgggctgcccttcctgg<br>ttggcttggtttcatcagccatccgcttgccctcatctgttacgccggcggtagccggccagcc<br>tcgcagagcaggattcccgttgagcaccgccaggtgcgaataagggacagtgaagaaggaacac<br>ccgctcgcgggtgggcctacttcacctatcctgcccggctgacgccgttggatacaccaaggaa<br>agtctacacgaaccctttggcaaaatcctgtatatcgtgcgaaaaaggatggatataccgaaaa<br>aatcgctataatgaccccgaagcagggttatgcagcggaaaagatccgtcgacctgctacgccg<br>ctacgtcttccgtgccgtcctgggcgtcgtcttcgtcgtcgtcggtcggcggcttcgcccacgt<br>gatcgaagcgcgcttctcgatgggcgttccctgccccctgcccgtagtcgacttcgtgacaacg<br>atcttgtctacgaagagcccgacgaacacgcgcttgtcgtctactgacgcgcgcccccaccacg<br>acttagggccggtcgggtcagcgtcggcgtcttcggggaaccattggtcaaggggaagcttcgg<br>ggcttcggcggcttcaagttcggcaagccgctcttccgccccttgctgccggagcgtcagcgct<br>gcctgttgcttccggaagtgcttcctgccaacgggtccgtcgtacgcgcctgccgcgcggtctt<br>cgtacagctcttcaagggcgttcagggcgtcggcgcgctccgcaacaaggttcgcccgttcgcc<br>gctcttctcaggcgcctcagtgagcttgccgaagcgtcgggcggcttcccacagaagcgccaac

		gtctcttcgtcgccttcggcgtgcctgatcttgttgaagatgcgttccgcaacgaacttgtcga<br>gtgccgccatgctgacgttgcacgtgccttcgtgctgcccaggtgcggacgggtcgaccacctt<br>ccggcgacggcagcggtaagagtccttgatcgattcttccccgcgcttcgaagtcatgacggcg<br>ccacactcgcagtacagcttgtccatggcggacagaatggcttgcccccgggaaagccccttgc<br>cgcgccccctgccgtccaaccacgcctgaagctcataccactcagcgggctcgatgatcggtcc<br>gcaatcaagctcgaccggccggagcgtgatcgggtcgcgctgaatgcggtaaccctcaatcttc<br>gtggtcggcgtgccgtccggcttcttcttgtagatcacctcagcggcgaagcccgcaatacgcg<br>ggtcccgaaggattcgcataacggttgccgggtcccaggcgcttgaagcggtcttcttcccaat<br>cgtctcgccccgggtcggcacggcgtcagcgtccatgcgcttacaaagccccgtgatgctgccc<br>gggtgaatggcggcttgactgcccggcttgaagggaaggtgtttgtgcgtcttgatctcacgcc<br>accaccaccggattacgtcgggctcgaactcgaagggtccggtaaggggagtggtcgagtgcgc<br>aagcttgttgatgacgacattgaccattcggccgttgcgcgtgatctccttcgtctccgaaaca<br>agctcgaagccgtaaggcgccttcccgccgacgtacccgcccaattcgcgctgaaggttcttcg<br>tgtcgagaatcttcgccgacttcagcgaagattctttgtgcgacgcgtcgagccgcataatcag<br>gtgaatcaggtccatgacgtttccctgccggaagacgccttcctgagtggaaacaatcgtcacg<br>cccagggcgagcaattccgagacaatcggaatcgcgtccatgaccttcaggcgcgagaagcgcg<br>acacgtcatagacaatgatcatgttgagccgcccggcgcggcattcgttcaggatgcgttcgaa<br>ctccgggcgctccgccgtcccgaacgccgacgtgcccggcgcttcgctgaaatgcccgacgaac<br>ctgaaccggcccccgtcgcgctcgacttcgcgctgaaggtcggccgccttgtcttcgttggcgc<br>tacgctgtgtcgctgggcttgctgcgctcgaactctcgcgctcgcgcgactgacggtcgtaagc<br>acccgcgtacgtgtccaccccggtcacaaccccttgtgtcatgtcggcgaccctacgcccccaa<br>ctgagagaactcaaaggttaccccagttggggcactactcccgaaaaccgcttctgacctggga<br>aaacgtgaagccccggggcatccgctgagggttgccgccggggcttcggtgtgtccgtcagtac<br>gggccatagagggggcgtcgtgtaacccacccagggcgattgctccggcacggggaagccgcgcc<br>acgccttcgggacgtctggaatcgctagagcttgcatgctgccacctgacgtctaagaagatcc<br>cgcaaaagcggcctttgactccctgcaagcctcagcgaccgaatatatcggttatgcgtgggcg<br>atggttgttgtcattgtcggcgcaactatcggtatcaagctgtttaagaaattcacctcgaaag<br>caagctgataaaccgatacaattaaaggctccttttggagccttttttttttggagattttcaac<br>gtgaaaaaattattattcgcaattcctttagttgttcctttctattctcactccgctgaaactg<br>ttgaaagttgtttagcaaaacctcatacagaaaattcatattaccctgttatccctactccgag<br>acagtcagagggtagaattcgcggccgcttctagagctcggtaccaaattccagaaaagaggcc<br>gcgaaagcggcctttttttcgttttggtcctactagatgcctccacaccgctcgtcacatcctgt<br>actagag
pABn	Plasmid backbone (Kan[R], *oriV*-based)[2]	tactagagctgcagcactcgttgctttatcggtatattaccctgttatccctaattttgttatc<br>aataaaaaaggcccccccgttagggaggccttattgttcgtcgctcactcaaaggcggtaatgcg<br>cgaattctgagctgccaggggtccccaataattacgatttaaattggcgaaaatgagacgttga<br>tcggcacgtaagaggttccaactttcaccataatgaaataagatcactaccgggcgtattttttt<br>gagttatcgagattttcaggagccacatttccccgaaaagtgccacctgggatgaatgtcagct<br>actgggctatctggacaagggaaaacgcaagcgcaaagagaaagcaggtagcttgcagtgggct<br>tacatggcgatagctagactgggcggttttatggacagcaagcgaaccggaattgccagctggg<br>gcgccctctggtaaggttgggaagccctgcaaagtaaactggatggctttcttgccgccaagga<br>tctgatggcgcaggggatcaagatctgatcaagagacaggatgaggatcgtttcgcATGATTGA<br>ACAAGATGGATTGCACGCAGGTTCTCCGGCCGCTTGGGTGGAGAGGCTATTCGGCTATGACTGG

		GCACAACAGACAATCGGCTGCTCTGATGCCGCCGTGTTCCGGCTGTCAGCGCAGGGGCGCCCGG<br>TTCTTTTTGTCAAGACCGACCTGTCCGGTGCCCTGAATGAACTGCAGGACGAGGCAGCGCGGCT<br>ATCGTGGCTGGCCACGACGGGCGTTCCTTGCGCAGCTGTGCTCGACGTTGTCACTGAAGCGGGA<br>AGGGACTGGCTGCTATTGGGCGAAGTGCCGGGGCAGGATCTCCTGTCATCTCACCTTGCTCCTG<br>CCGAGAAAGTATCCATCATGGCTGATGCAATGCGGCGGCTGCATACGCTTGATCCGGCTACCTG<br>CCCATTCGACCACCAAGCGAAACATCGCATCGAGCGAGCACGTACTCGGATGGAAGCCGGTCTT<br>GTCGATCAGGATGATCTGGACGAAGAGCATCAGGGGCTCGCGCCAGCCGAACTGTTCGCCAGGC<br>TCAAGGCGCGCATGCCCGACGGCGAGGATCTCGTCGTGACCCATGGCGATGCCTGCTTGCCGAA<br>TATCATGGTGGAAAATGGCCGCTTTTCTGGATTCATCGACTGTGGCCGGCTGGGTGTGGCGGAC<br>CGCTATCAGGACATAGCGTTGGCTACCCGTGATATTGCTGAAGAGCTTGGCGGCGAATGGGCTG<br>ACCGCTTCCTCGTGCTTTACGGTATCGCCGCTCCCGATTCGCAGCGCATCGCCTTCTATCGCCT<br>TCTTGACGAGTTCTTCTGAtttgacttttgtccttttccgctgcataaccctgcttcggggtca<br>ttatagcgatttttttcggtatatccatcctttttcgcacgatatacaggattttgccaaagggt<br>tcgtgtagactttccttggtgtatccaacggcgtcagccgggcaggataggtgaagtaggccca<br>cccgcgagcgggtgttccttcttcactgtcccttattcgcacctggcggtgctcaacgggaatc<br>ctgctctgcgaggctggccgtaggccggccgcgatgcaggtggctgctgaacccccagccggaa<br>ctgaccccacaaggccctagcgtttgcaatgcaccaggtcatcattgacccaggcgtgttccac<br>caggccgctgcctcgcaactcttcgcaggcttcgccgacctgctcgcgccacttcttcacgcgg<br>gtggaatccgatccgcacatgaggcggaaggtttccagcttgagcgggtacggctcccggtgcg<br>agctgaaatagtcgaacatccgtcgggccgtcggcgacagcttgcggtacttctcccatatgaa<br>tttcgtgtagtggtcgccagcaaacagcacgacgatttcctcgtcgatcaggacctggcaacgg<br>gacgttttcttgccacggtccaggacgcggaagcggtgcagcagcgacaccgattccaggtgcc<br>caacgcggtcggacgtgaagcccatcgccgtcgcctgtaggcgcgacaggcattcctcggcctt<br>cgtgtaataccggccattgatcgaccagcccaggtcctggcaaagctcgtagaacgtgaaggtg<br>atcggctcgccgataggggtgcgcttcgcgtactccaacacctgctgccacaccagttcgtcat<br>cgtcggcccgcagctcgacgccggtgtaggtgatcttcacgtccttgttgacgtggaaaatgac<br>cttgttttgcagcgcctcgcgcgggattttcttgttgcgcgtggtgaacagggcagagcgggcc<br>gtgtcgtttggcatcgctcgcatcgtgtccggccacggcgcaatatcgaacaaggaaagctgca<br>tttccttgatctgctgcttcgtgtgtttcagcaacgcggcctgcttggcttcgctgacctgttt<br>tgccaggtcctcgccggcggtttttcgcttcttggtcgtcatagttcctcgcgtgtcgatggtc<br>atcgacttcgccaaacctgccgcctcctgttcgagacgacgcgaacgctccacggcggccgatg<br>gcgcgggcagggcagggggagccagttgcacgctgtcgcgctcgatcttggccgtagcttgctg<br>gactatcgagccgacggactggaaggtttcgcggggcgcacgcatgacggtgcggcttgcgatg<br>gtttcggcatcctcggcggaaaaccccgcgtcgatcagttcttgcctgtatgccttccggtcaa<br>acgtccgattcattcaccctccttgcgggattgccccggaattaattccccggatcgatccgtc<br>gatcttgatcccctgcgccatcagatccttggcggcaagaaagccatccagtttactttgcagg<br>gcttcccaaccttaccagagggcgccccagctggcaattccggttcgcttgctgtccataaaac<br>cgcccagtctagctatcgccatgtaagcccactgcaagctacctgctttctctttgcgcttgcg<br>ttttcccttgtccagatagcccagtagctgacattcatccggggtcagcaccgtttctgcggac<br>tggctttctacgtggctgccatttttggggtgaggccgttcgcggccgaggggcgcagcccctg<br>gggggatgggaggcccgcgttagcgggccgggagggttcgagaagggggggcacccccttcgg<br>cgtgcgcggtcacgcgcacagggcgcagccctggttaaaaacaaggtttataaatattggttta<br>aaagcaggttaaaagacaggttagcggtggccgaaaaacgggcggaaacccttgcaaatgctgg

		attttctgcctgtggacagcccctcaaatgtcaataggtgcgcccctcatctgtcagcactctg<br>cccctcaagtgtcaaggatcgcgcccctcatctgtcagtagtcgcgcccctcaagtgtcaatac<br>cgcagggcacttatccccaggcttgtccacatcatctgtgggaaactcgcgtaaaatcaggcgt<br>tttcgccgatttgcgaggctggccagctccacgtcgccggccgaaatcgagcctgcccctcatc<br>tgtcaacgccgcgccgggtgagtcggcccctcaagtgtcaacgtccgcccctcatctgtcagtg<br>agggccaagttttccgcgaggtatccacaacgccggcggccctacatggctctgctgtagtgag<br>tgggttgcgctccggcagcggtcctgatccccgcagaaaaaaaggatctcaagaagatccttt<br>gatcttttctacggcgcgcccagctgtctagggcggcggatttgtcctactcaggagagcgttc<br>accgacaaacaacagataaaacgaaaggcccagtctttcgactgagcctttcgttttatttgat<br>gcctttaattaaagcggataacaatttcacacaggaggccgtgccacctgacgtctaagaaaag<br>gaatattcagcaatttgcccgtgccgaagaaaggcccacccgtgaaggtgagccagtgagttga<br>ttgctacgtaattagttagttagcccttagtgactcgaattc**tttacggctagctcagtcctag**<br>**gtatagtgctagctactagagaaagaggagaaatactagatggtaataATGAGCCCAAAAAGAA**<br>**GAACACAAGCAGAAAGAGCAATGGAAACACAAGGAAAACTAATAGCAGCAGCACTAGGAGTACT**<br>**AAGAGAAAAAGGATACGCAGGATTCAGAATAGCAGACGTACCAGGAGCAGCAGGAGTAAGCAGA**<br>**GGAGCACAAAGCCACCACTTCCCAACAAAACTAGAACTACTACTAGCAACATTCGAATGGCTAT**<br>**ACGAACAAATAACAGAAAGAAGCAGAGCAAGACTAGCAAAACTAAAACCAGAGGACGACGTAAT**<br>**ACAACAAATGCTAGACGACGCAGCAGAGTTCTTCCTAGACGACGACTTCAGCATAAGCCTAGAC**<br>**CTAATAGTAGCAGCAGACCGCGACCCAGCACTAAGAGAAGGAATACAAAGAACAGTAGAAAGAA**<br>**ACAGATTCGTAGTAGAGGACATGTGGCTAGGAGTGCTAGTAAGCAGAGGACTAAGCAGAGACGA**<br>**CGCAGAGGACATACTATGGCTAATATTCAACAGCGTAAGAGGACTAGCAGTAAGAAGCCTATGG**<br>**CAAAAAGACAAAGAAAGATTCGAAAGAGTAAGAAACAGCACACTAGAAATAGCAAGAGAAAGAT**<br>**ACGCAAAATTCAAAAGATAAtaactcggtaccaaattccagaaaagaggcctcccgaaagggggg**<br>**gcctttttttcgttttggtcc**tactagaggttatgagtcaggaaaaaaggcgacagagtaatctg<br>tcgcctttttttctttgcttgcttttactagag
pAPn	Plasmid backbone (Amp^R, *p15A & pRO1600 V*-based)[3]	tttatactgcaggatttgaacgttgcgaagcaacggcccggagggtggcgggcaggacgcccgc<br>cataaactgccaggcatcaaattaagcagaaggccatcctgacggatggcctttttgcgtttcg<br>aacaattgaaaaaacctcgcgccttacctgttgagtaatagtcaaaagcctccggtcggaggct<br>tttgactttctgcttactgaatttcggtggtgccgttactaggccagatccagcggcatctggg<br>ttagtcgagcgcgggccgcttcccatgtctcaccagggcgagcctgtttcgcgatctcagcatc<br>tgaaatcttcccggccttgcgcttcgctggggccttacccaccgccttggcgggcttcttcggt<br>ccaaaactgaacaacagatgtgtgaccttgcgcccggtctttcgctgcgcccactccacctgta<br>gcgggctgtgctcgttgatctgcgtcacggctggatcaagcactcgcaacttgaagtccttgat<br>cgagggataccggccttccagttgaaaccactttcgcagctggtcaatttctatttcgcgctgg<br>ccgatgctgtcccattgcatgagcagctcgtaaagcctgatcgcgtgggtgctgtccatcttgg<br>ccacgtcagccaaggcgtatttggtgaactgtttggtgagttccgtcaggtacggcagcatgtc<br>tttggtgaacctgagttctacacggccctcaccctcccggtagatgattgtttgcacccagccg<br>gtaatcatcacactcggtcttttccccttgccattgggctcttgggttaaccggacttcccgcc<br>gtttcaggcgcagggccgcttctttgagctggttgtaggaagattcgatagggacacccgccat<br>cgtcgctatgtcctccgccgtcactgaatacatcacttcatcggtgacaggctcgctcctcttc<br>acctggctaatacaggccagaacgatccgctgttcctgaacactgaggcgatacgcggcctcga<br>ccagggcattgcttttgtaaaccattggggtgaggccacgttcgacattccttgtgtataagg<br>ggacactgtatctgcgtcccacaatacaacaaatccgtccctttacaacaacaaatccgtccct

		tcttaacaacaaatccgtcccttaatggcaacaaatccgtccctttttaaactctacaggccac<br>ggattacgtggcctgtagacgtcctaaaaggtttaaaagggaaaaggaagaaaagggtggaaac<br>gcaaaaaacgcaccactacgtggccccgttggggccgcatttgtgcccctgaaggggcgggga<br>ggcgtctgggcaatccccgttttaccagtcccctatcgccgcctgagagggcgcaggaagcgag<br>taatcagggtatcgaggcggattcacccttggcgtccaaccagcggcaccagcggcgcctgaga<br>ggatggtgcactctcagtacaatctgctctgatgccgcatagttaagccagccccgacacccgc<br>caacacccgctgacgcgccctgacgggcttgtctgctcccggcatccgcttacagacaagctgt<br>gaccgtctccgggagctgcatgtgtcagaggttttcaccgtcatcaccgaaacgcgcgagacga<br>aagggcctcgtgatacgcctatttttataggttaatgtcatgataataatggtttcttagacgt<br>caggtggcacttttcggggaaatgtgcgcggaacccctatttgtttatttttctaaatacattc<br>aaatatgtatccgctcatgagacaataaccctgataaatgcttcaataatattgaaaaaggaag<br>actATGAGTATTCAACATTTCCGTGTCGCCCTTATTCCCTTTTTTGCGGCATTTTGCCTTCCTG<br>TTTTTGCTCACCCAGAAACGCTGGTGAAAGTAAAAGATGCTGAAGATCAGTTGGGTGCACGAGT<br>GGGTTACATCGAACTGGATCTCAACAGCGGTAAGATCCTTGAGAGTTTTCGCCCCGAAGAACGT<br>TTTCCAATGATGAGCACTTTTAAAGTTCTGCTATGTGGCGCGGTATTATCCCGTGTTGACGCCG<br>GGCAAGAGCAACTCGGTCGCCGCATACACTATTCTCAGAATGACTTGGTTGAGTACTCACCAGT<br>CACAGAAAAGCATCTTACGGATGGCATGACAGTAAGAGAATTATGCAGTGCTGCCATAACCATG<br>AGTGATAACACTGCGGCCAACTTACTTCTGACAACGATCGGAGGACCGAAGGAGCTAACCGCTT<br>TTTTGCACAACATGGGGGATCATGTAACTCGCCTTGATCGTTGGGAACCGGAGCTGAATGAAGC<br>CATACCAAACGACGAGCGTGACACCACGATGCCTGCAGCAATGGCAACAACGTTGCGCAAACTA<br>TTAACTGGCGAACTACTTACTCTAGCTTCCCGGCAACAATTAATAGACTGGATGGAGGCGGATA<br>AAGTTGCAGGACCACTTCTGCGCTCGGCCCTTCCGGCTGGCTGGTTTATTGCTGATAAATCTGG<br>AGCCGGTGAGCGTGGATCTCGCGGTATCATTGCAGCACTGGGGCCAGATGGTAAGCCCTCCCGT<br>ATCGTAGTTATCTACACGACGGGGAGTCAGGCAACTATGGATGAACGAAATAGACAGATCGCTG<br>AGATAGGTGCCTCACTGATTAAGCATTGGTAActgtcagaccaagtttactcatatatacttta<br>gattgatttacgcgccctgtagcggcgcattaagcgcggcgggtgtggtggttacgcgcagcgt<br>gaccgctacacttgccagcgccctagcgcccgctcctttcgctttcttcccttcctttctcgcc<br>acgttcgccggctttccccgtcaagctctaaatcgggggctccctttagggttccgatttagtg<br>ctttacggcacctcgaccccaaaaaacttgatttgggtgatggttcacgtagtgggccatcgcc<br>ctgatagacggtttttcgccctttgacgttggagtccacgttctttaatagtggactcttgttc<br>caaacttgaacaacactcaaccctatctcgggctattcttttgatttataagggattttgccga<br>tttcggcctattggttaaaaaatgagctgatttaacaaaaatttaacgcgaattttaacaaaat<br>attaacgtttacaatttaaaaggatctaggtgaagatcctttttgataatctcatgaccaaaat<br>cccttaacgtgagttttcgttccactgagcgtcagaccccgtagaaaagatcaaaggatcttct<br>tgagatcctttttttctgcgcgtaatctgctgcttgcaaacaaaaaaaccaccgctaccagcgg<br>tggtttgtttgccggatcaagagctaccaactctttttccgaaggtaactggcttcagcagagc<br>gcagataccaaatactgtccttctagtgtagccgtagttaggccaccacttcaagaactctgta<br>gcaccgcctacatacctcgctctgctaatcctgttaccagtcaggcatttgagaagcacacggt<br>cacactgcttccggtagtcaataaaccggtaaaccagcaatagacataagcggctatttaacga<br>ccctgccctgaaccgacgaccgggtcgaatttgctttcgaatttctgccattcatccgcttatt<br>atcacttattcaggcgtagcaccaggcgtttaagggcaccaataactgccttaaaaaaattacg<br>ccccgccctgccactcatcgcagtactgttgtaattcattaagcattctgccgacatggaagcc<br>atcacagacggcatgatgaacctgaatcgccagcggcatcagcaccttgtcgccttgcgtataa

		tatttgcccatggctagcggagtgtatactggcttactatgttggcactgatgagggtgtcagt<br>gaagtgcttcatgtggcaggagaaaaaaggctgcaccggtgcgtcagcagaatatgtgatacag<br>gatatattccgcttcctcgctcactgactcgctacgctcggtcgttcgactgcggcgagcggaa<br>atggcttacgaacggggcggagatttcctggaagatgccaggaagatacttaacagggaagtga<br>gagggccgcggcaaagccgtttttccataggctccgcccccctgacaagcatcacgaaatctga<br>cgctcaaatcagtggtggcgaaacccgacaggactataaagataccaggcgtttccccctggcg<br>gctccctcgtgcgctctcctgttcctgcctttcggtttaccggtgtcattccgctgttatggcc<br>gcgtttgtctcattccacgcctgacactcagttccgggtaggcagttcgctccaagctggactg<br>tatgcacgaaccccccgttcagtccgaccgctgcgccttatccggtaactatcgtcttgagtcc<br>aacccggaaagacatgcaaaagcaccactggcagcagccactggtaattgatttagaggagtta<br>gtcttgaagtcatgcgccggttaaggctaaactgaaaggacaagttttggtgactgcgctcctc<br>caagccagttacctcggttcaaagagttggtagctcagagaaccttcgaaaaaccgccctgcaa<br>ggcggttttttcgttttcagagcaagagattacgcgcagaccaaaacgatctcaagaagatcat<br>cttattaatcagataaaatatttgctcatgagcccgaagtggcgagcccgatcttccccatcgg<br>tgatgtcggcgatataggcgccagcaaccgcacctgtggcgccggtgatgccggccacgatgcg<br>tccggcgtagaggatctgctcatgtttgacagcttatcatcgatgcataatgtgcctgtcaaat<br>ggacgaagcagggattctgcaaaccctatgctactccctcgagccgtcaattgtctgattcgtt<br>accaattagaattc**TCACTGCCCGCTTTCCAGTCGGGAAACCTGTCGTGCCAGCTGCATTAATG**<br>**AATCGGCCAACGCGCGGGGAGAGGCGGTTTGCGTATTGGGCGCCAGGGTGGTTTTTCTTTTCAC**<br>**CAGTGAGACTGGCAACAGCTGATTGCCCTTCACCGCCTGGCCCTGAGAGAGTTGCAGCAAGCGG**<br>**TCCACGCTGGTTTGCCCCAGCAGGCGAAAATCCTGTTTGATGGTGGTTAACGGCGGGATATAAC**<br>**ATGAGCTATCTTCGGTATCGTCGTATCCCACTACCGAGATATCCGCACCAACGCGCAGCCCGGA**<br>**CTCGGTAATGGCGCGCATTGCGCCCAGCGCCATCTGATCGTTGGCAACCAGCATCGCAGTGGGA**<br>**ACGATGCCCTCATTCAGCATTTGCATGGTTTGTTGAAAACCGGACATGGCACTCCAGTCGCCTT**<br>**CCCGTTCCGCTATCGGCTGAATTTGATTGCGAGTGAGATATTTATGCCAGCCAGCCAGACGCAG**<br>**ACGCGCCGAGACAGAACTTAATGGGCCCGCTAACAGCGCGATTTGCTGGTGACCCAATGCGACC**<br>**AGATGCTCCACGCCCAGTCGCGTACCGTCCTCATGGGAGAAAATAATACTGTTGATGGGTGTCT**<br>**GGTCAGAGACATCAAGAAATAACGCCGGAACATTAGTGCAGGCAGCTTCCACAGCAATGGCATC**<br>**CTGGTCATCCAGCGGATAGTTAATGATCAGCCCACTGACGCGTTGCGCGAGAAGATTGTGCACC**<br>**GCCGCTTTACAGGCTTCGACGCCGCTTCGTTCTACCATCGACACCACCACGCTGGCACCCAGTT**<br>**GATCGGCGCGAGATTTAATCGCCGCGACAATTTGCGACGGCGCGTGCAGGGCCAGACTGGAGGT**<br>**GGCAACGCCAATCAGCAACGACTGTTTGCCCGCCAGTTGTTGTGCCACGCGGTTGGGAATGTAA**<br>**TTCAGCTCCGCCATCGCCGCTTCCACTTTTTCCCGCGTTTTCGCAGAAACGTGGCTGGCCTGGT**<br>**TCACCACGCGGGAAACGGTCTGATAAGAGACACCGGCATACTCTGCGACATCGTATAACGTTAC**<br>**TGGTTTCAT**attcaccaccctgaattgactctcttccgggcgctatcatgccataccgcgaaag<br>gttttgcgccattcgatggcgcgccgcttcgtcaggccacatagctttcttgttctgatcggaa<br>cgatcgttggctgctcgagccgtcaattgtctgattcgttaccaattagaattcctcggtacca<br>aattccagaaaagaggcctcccgaaagggggcctttttttcgttttggtcctactagaggttat<br>gagtcaggaaaaaaggcgacagagtaatctgtcgcctttttttctttgcttgcttttactagag

[1] For each plasmid backbone, the replicon sequence was shown with underlines and the resistant gene CDS was indicated in capital letters.

[2] The CymR-related cassette was shown in bold and the *cymR* CDS was indicated in capital letters.

[3] The *lacI* CDS was shown in bold and indicated in capital letters.

Details of Parts Substitutions

In order to define all the components used in this project in an orderly manner, an initial version of the genetic circuit needs to be preset. For convenience, we define the RPA circuit that first presents the time-course data in the main manuscript (Figure 2B) as the initial version (RPA v1.0). the construction details of this circuit were summarized in Supplementary Table 2, and all other part substitutions for the specific parameter perturbation and topology damage experiments were summarized in Supplementary Table 3.

Supplementary Table 2

Critical parts composition for RPA v1.0 circuit[1].

Based Plasmids	Part Num	Function	Sequence (5' - 3')[2]
pAR	I	pT_7wt[3]	TAATACGACTCACTATAGGGG
	II	CymR operator	AACAAACAGACAATCTGGTCTGTTTGTA~~TTTACTAGAG~~
	III	gRNA region[4]	GTCTAAGAACTTTAAATAATTTCTACTGTTGTAGATGCTCGCGAACAGTTGGCC<br>CTGTCTAAGAACTTTAAATAATTTCTACTGTTGTAGATAGCGTCAACTTAAAGC<br>TGGTGTCTAAGAACTTTAAATAATTTCTACTGTTGTAGATAGGAAGTGAAAGCT<br>AAGCGCGTCTAAGAACTTTAAATAATTTCTACTGTTGTAGAT
	IV	Ribozyme-based insulator	AGCTGTCACCGGATGTGCTTTCCGGTCTGATGAGTCCGTGAGGACGAAACAGCC<br>TCTACAAATAATTTTGTTTAA
	V	RBS	ATTTAAATTCTTTTAAGGAGTAAGT
	VI	sfGFP reporter	ATGCGTAAAGGCGAAGAGCTGTTCACTGGTGTCGTCCCTATTCTGGTGGAACTG<br>GATGGTGATGTCAACGGTCATAAGTTTTCCGTGCGTGGCGAGGGTGAAGGTGAC<br>GCAACTAATGGTAAACTGACGCTGAAGTTCATCTGTACTACTGGTAAACTGCCG<br>GTACCTTGGCCGACTCTGGTAACGACGCTGACTTATGGTGTTCAGTGCTTTGCT<br>CGTTATCCGGACCATATGAAGCAGCATGACTTCTTCAAGTCCGCCATGCCGGAA<br>GGCTATGTGCAGGAACGCACGATTTCCTTTAAGGATGACGGCACGTACAAAACG<br>CGTGCGGAAGTGAAATTTGAAGGCGATACCCTGGTAAACCGCATTGAGCTGAAA<br>GGCATTGACTTTAAAGAAGACGGCAATATCCTGGGCCATAAGCTGGAATACAAT<br>TTTAACAGCCACAATGTTTACATCACCGCCGATAAACAAAAAAATGGCATTAAA<br>GCGAATTTTAAAATTCGCCACAACGTGGAGGATGGCAGCGTGCAGCTGGCTGAT<br>CACTACCAGCAAAACACTCCAATCGGTGATGGTCCTGTTCTGCTGCCAGACAAT<br>CACTATCTGAGCACGCAAAGCGTTCTGTCTAAAGATCCGAACGAGAAACGCGAT<br>CATATGGTTCTGCTGGAGTTCGTAACCGCAGCGGGCATCACGCATGGTATGGAT<br>GAACTGTACAAATGA
	VII	Composite Terminator[5]	~~TGATAAGCCAGGCAT~~CAAATAAAACGAAAGGCTCAGTCGAAAGACTGGGCCTTT<br>CGTTTTATCTGTTGTTTGTCGGTGAACGCTCTC~~TACTAGAGTCACACT~~GGCTCA<br>CCTTCGGGTGGGCCTTTCTGCG
pAP	I	pT_7wt[3]	TAATACGACTCACTATAGGGG

	II	CymR operator	AACAAACAGACAATCTGGTCTGTTTGTATTT~~ACTAGAG~~
	III	Ribozyme-based insulator	AGCTGTCACCGGATGTGCTTTCCGGTCTGATGAGTCCGTGAGGACGAAACAGCC<br>TCTACAAATAATTTTGTTTAA
	IV	RBS	AGATTTTAAGGGGGGTAAGGATAT
	V	FndCpf1	ATGTCAATTTATCAAGAATTTGTTAATAAATATAGTTTAAGTAAAACTCTAAGA<br>TTTGAGTTAATCCCACAGGGTAAAACACTTGAAAACATAAAAGCAAGAGGTTTG<br>ATTTTAGATGATGAGAAAAGAGCTAAAGACTACAAAAAGGCTAAACAAATAATT<br>GATAAATATCATCAGTTTTTTATAGAGGAGATATTAAGTTCGGTTTGTATTAGC<br>GAAGATTTATTACAAAACTATTCTGATGTTTATTTTAAACTTAAAAAGAGTGAT<br>GATGATAATCTACAAAAAGATTTTAAAAGTGCAAAAGATACGATAAAGAAACAA<br>ATATCTGAATATATAAAGGACTCAGAGAAATTTAAGAATTTGTTTAATCAAAAC<br>CTTATCGATGCTAAAAAAGGGCAAGAGTCAGATTTAATTCTATGGCTAAAGCAA<br>TCTAAGGATAATGGTATAGAACTATTTAAAGCCAATAGTGATATCACAGATATA<br>GATGAGGCGTTAGAAATAATCAAATCTTTTAAAGGTTGGACAACTTATTTTAAG<br>GGTTTTCATGAAAATAGAAAAAATGTTTATAGTAGCAATGATATTCCTACATCT<br>ATTATTTATAGGATAGTAGATGATAATTTGCCTAAATTTCTAGAAAATAAAGCT<br>AAGTATGAGAGTTTAAAAGACAAAGCTCCAGAAGCTATAAACTATGAACAAATT<br>AAAAAAGATTTGGCAGAAGAGCTAACCTTTGATATTGACTACAAAACATCTGAA<br>GTTAATCAAAGAGTTTTTTCACTTGATGAAGTTTTTGAGATAGCAAACTTTAAT<br>AATTATCTAAATCAAAGTGGTATTACTAAATTTAATACTATTATTGGTGGTAAA<br>TTTGTAAATGGTGAAAATACAAAGAGAAAAGGTATAAATGAATATATAAATCTA<br>TACTCACAGCAAATAAATGATAAAACACTCAAAAAATATAAAATGAGTGTTTTA<br>TTTAAGCAAATTTTAAGTGATACAGAATCTAAATCTTTTGTAATTGATAAGTTA<br>GAAGATGATAGTGATGTAGTTACAACGATGCAAAGTTTTTATGAGCAAATAGCA<br>GCTTTTAAAACAGTAGAAGAAAAATCTATTAAAGAAACACTATCTTTATTATTT<br>GATGATTTAAAAGCTCAAAAACTTGATTTGAGTAAAATTTATTTTAAAAATGAT<br>AAATCTCTTACTGATCTATCACAACAAGTTTTTGATGATTATAGTGTTATTGGT<br>ACAGCGGTACTAGAATATATAACTCAACAAATAGCACCTAAAAATCTTGATAAC<br>CCTAGTAAGAAAGAGCAAGAATTAATAGCCAAAAAAACTGAAAAAGCAAAATAC<br>TTATCTCTAGAAACTATAAAGCTTGCCTTAGAAGAATTTAATAAGCATAGAGAT<br>ATAGATAAACAGTGTAGGTTTGAAGAAATACTTGCAAACTTTGCGGCTATTCCG<br>ATGATATTTGATGAAATAGCTCAAAACAAAGACAATTTGGCACAGATATCTATC<br>AAATATCAAAATCAAGGTAAAAAAGACCTACTTCAAGCTAGTGCGGAAGATGAT<br>GTTAAAGCTATCAAGGATCTTTTAGATCAAACTAATAATCTCTTACATAAACTA<br>AAAATATTTCATATTAGTCAGTCAGAAGATAAGGCAAATATTTTAGACAAGGAT<br>GAGCATTTTTATCTAGTATTTGAGGAGTGCTACTTTGAGCTAGCGAATATAGTG<br>CCTCTTTATAACAAAATTAGAAACTATATAACTCAAAAGCCATATAGTGATGAG<br>AAATTTAAGCTCAATTTTGAGAACTCGACTTTGGCTAATGGTTGGGATAAAAAT<br>AAAGAGCCTGACAATACGGCAATTTTATTTATCAAAGATGATAAATATTATCTG<br>GGTGTGATGAATAAGAAAAATAACAAAATATTTGATGATAAAGCTATCAAAGAA<br>AATAAAGGCGAGGGTTATAAAAAAATTGTTTATAAACTTTTACCTGGCGCAAAT

			AAAATGTTACCTAAGGTTTTCTTTTCTGCTAAATCTATAAAATTTTATAATCCT<br>AGTGAAGATATACTTAGAATAAGAAATCATTCCACACATACAAAAAATGGTAGT<br>CCTCAAAAAGGATATGAAAAATTTGAGTTTAATATTGAAGATTGCCGAAAATTT<br>ATAGATTTTTATAAACAGTCTATAAGTAAGCATCCGGAGTGGAAAGATTTTGGA<br>TTTAGATTTTCTGATACTCAAAGATATAATTCTATAGATGAATTTTATAGAGAA<br>GTTGAAAATCAAGGCTACAAACTAACTTTTGAAAATATATCAGAGAGCTATATT<br>GATAGCGTAGTTAATCAGGGTAAATTGTACCTATTCCAAATCTATAATAAAGAT<br>TTTTCAGCTTATAGCAAAGGGCGACCAAATCTACATACTTTATATTGGAAAGCG<br>CTGTTTGATGAGAGAAATCTTCAAGATGTGGTTTATAAGCTAAATGGTGAGGCA<br>GAGCTTTTTTATCGTAAACAATCAATACCTAAAAAAATCACTCACCCAGCTAAA<br>GAGGCAATAGCTAATAAAAACAAAGATAATCCTAAAAAAGAGAGTGTTTTTGAA<br>TATGATTTAATCAAAGATAAACGCTTTACTGAAGATAAGTTTTTCTTTCACTGT<br>CCTATTACAATCAATTTTAAATCTAGTGGAGCTAATAAGTTTAATGATGAAATC<br>AATTTATTGCTAAAAGAAAAAGCAAATGATGTTCATATATTAAGTATAGCAAGA<br>GGTGAAAGACATTTAGCTTACTATACTTTGGTAGATGGTAAAGGCAATATCATC<br>AAACAAGATACTTTCAACATCATTGGTAATGATAGAATGAAAACAAACTACCAT<br>GATAAGCTTGCTGCAATAGAGAAAGATAGGGATTCAGCTAGGAAAGACTGGAAA<br>AAGATAAATAACATCAAAGAGATGAAAGAGGGCTATCTATCTCAGGTAGTTCAT<br>GAAATAGCTAAGCTAGTTATAGAGTATAATGCTATTGTGGTTTTTGAGGATTTA<br>AATTTTGGATTTAAAAGAGGGCGTTTCAAGGTAGAGAAGCAGGTCTATCAAAAG<br>TTAGAAAAAATGCTAATTGAGAAACTAAACTATCTAGTTTTCAAAGATAATGAG<br>TTTGATAAAACTGGGGGAGTGCTTAGAGCTTATCAGCTAACAGCACCTTTTGAG<br>ACTTTTAAAAAGATGGGTAAACAAACAGGTATTATCTACTATGTACCAGCTGGT<br>TTTACTTCAAAAATTTGTCCTGTAACTGGTTTTGTAAATCAGTTATATCCTAAG<br>TATGAAAGTGTCAGCAAATCTCAAGAGTTCTTTAGTAAGTTTGACAAGATTTGT<br>TATAACCTTGATAAGGGCTATTTTGAGTTTAGTTTTGATTATAAAAACTTTGGT<br>GACAAGGCTGCCAAAGGCAAGTGGACTATAGCTAGCTTTGGGAGTAGATTGATT<br>AACTTTAGAAATTCAGATAAAAATCATAATTGGGATACTCGAGAAGTTTATCCA<br>ACTAAAGAGTTGGAGAAATTGCTAAAAGATTATTCTATCGAATATGGGCATGGC<br>GAATGTATCAAAGCAGCTATTTGCGGTGAGAGCGACAAAAAGTTTTTTGCTAAG<br>CTAACTAGTGTCCTAAATACTATCTTACAAATGCGTAACTCAAAAACAGGTACT<br>GAGTTAGATTATCTAATTTCACCAGTAGCAGATGTAAATGGCAATTTCTTTGAT<br>TCGCGACAGGCGCCAAAAAATATGCCTCAAGATGCTGATGCCAATGGTGCTTAT<br>CATATTGGGCTAAAAGGTCTGATGCTACTAGGTAGGATCAAAAATAATCAAGAG<br>GGCAAAAAACTCAATTTGGTTATCAAAAATGAAGAGTATTTTGAGTTCGTGCAG<br>AATAGGAATAACTAG
	VI	Composite Terminator[5]	~~TGATAAGCCAGCA~~TCAATAAAACGAAAGGCTCAGTCGAAAGACTGGGCCTTTCG<br>TTTTATCTGTTGTTTGTCGGTGAACGCTCTC~~TACTAGAGTCACACT~~GGCTCACC<br>TTCGGGTGGGCCTTTCTGCG
pBB	I	pT_7m_3[3]	GGATACGACTCACTATAGGGG~~TACTAGAG~~
	II	Ribozyme-based insulator	AGTACGTCTGAGCGTGATACCCGCTCACTGAAGATGGCCCGGTAGGGCCGAAAC<br>GTACCTCTACAAATAATTTTGTTTAA

	III	RBS	~~TACTAGAG~~GCCTCTCGTTAC~~TACTAG~~
	IV	T_7 RNAP[4]	ATGAACACGATTAACATCGCTAAGAACGACTTCTCTGACATCGAACTGGCTGCT<br>ATCCCGTTCAACACTCTGGCTGACCATTACGGTGAGCGTTTA**GCTCGCGAACAG**<br>**TTGGCCCT**TGAGCATGAGTCTTACGAGATGGGTGAAGCACGCTTCCGCAAGATG<br>TTTG**AGCGTCAACTTAAAGCTGGT**GAGGTTGCGGATAACGCTGCCGCCAAGCCT<br>CTCATCACTACCCTACTCCCTAAGATGATTGCACGCATCAACGACTGGTTTG**AG**<br>**GAAGTGAAAGCTAAGCGC**GGCAAGCGCCCGACAGCCTTCCAGTTCCTGCAAGAA<br>ATCAAGCCGGAAGCCGTAGCGTACATCACCATTAAGACCACTCTGGCTTGCCTA<br>ACCAGTGCTGACAATACAACCGTTCAGGCTGTAGCAAGCGCAATCGGTCGGGCC<br>ATTGAGGACGAGGCTCGCTTCGGTCGTATCCGTGACCTTGAAGCTAAGCACTTC<br>AAGAAAAACGTTGAGGAACAACTCAACAAGCGCGTAGGGCACGTCTACAAGAAA<br>GCATTTATGCAAGTTGTCGAGGCTGACATGCTCTCTAAGGGTCTACTCGGTGGC<br>GAGGCGTGGTCTTCGTGGCATAAGGAAGACTCTATTCATGTAGGAGTACGCTGC<br>ATCGAGATGCTCATTGAGTCAACCGGAATGGTTAGCTTACACCGCCAAAATGCT<br>GGCGTAGTAGGTCAAGACTCTGAGACTATCGAACTCGCACCTGAATACGCTGAG<br>GCTATCGCAACCCGTGCAGGTGCGCTGGCTGGCATCTCTCCGATGTTCCAACCT<br>TGCGTAGTTCCTCCTAAGCCGTGGACTGGCATTACTGGTGGTGGCTATTGGGCT<br>AACGGTCGTCGTCCTCTGGCGCTGGTGCGTACTCACAGTAAGAAAGCACTGATG<br>CGCTACGAAGACGTTTACATGCCTGAGGTGTACAAAGCGATTAACATTGCGCAA<br>AACACCGCATGGAAAATCAACAAGAAAGTCCTAGCGGTCGCCAACGTAATCACC<br>AAGTGGAAGCATTGTCCGGTCGAGGACATCCCTGCGATTGAGCGTGAAGAACTC<br>CCGATGAAACCGGAAGACATCGACATGAATCCTGAGGCTCTCACCGCGTGGAAA<br>CGTGCTGCCGCTGCTGTGTACCGCAAGGACAAGGCTCGCAAGTCTCGCCGTATC<br>AGCCTTGAGTTCATGCTTGAGCAAGCCAATAAGTTTGCTAACCATAAGGCCATC<br>TGGTTCCCTTACAACATGGACTGGCGCGGTCGTGTTTACGCTGTGTCAATGTTC<br>AACCCGCAAGGTAACGATATGACCAAAGGACTGCTTACGCTGGCGAAAGGTAAA<br>CCAATCGGTAAGGAAGGTTACTACTGGCTGAAAATCCACGGTGCAAACTGTGCG<br>GGTGTCGATAAGGTTCCGTTCCCTGAGCGCATCAAGTTCATTGAGGAAAACCAC<br>GAGAACATCATGGCTTGCGCTAAGTCTCCACTGGAGAACACTTGGTGGGCTGAG<br>CAAGATTCTCCGTTCTGCTTCCTTGCGTTCTGCTTTGAGTACGCTGGGGTACAG<br>CACCACGGCCTGAGCTATAACTGCTCCCTTCCGCTGGCGTTTGACGGGTCTTGC<br>TCTGGCATCCAGCACTTCTCCGCGATGCTCCGAGATGAGGTAGGTGGTCGCGCG<br>GTTAACTTGCTTCCTAGTGAAACCGTTCAGGACATCTACGGGATTGTTGCTAAG<br>AAAGTCAACGAGATTCTACAAGCAGACGCAATCAATGGGACCGATAACGAAGTA<br>GTTACCGTGACCGATGAGAACACTGGTGAAATCTCTGAGAAAGTCAAGCTGGGC<br>ACTAAGGCACTGGCTGGTCAATGGCTGGCTTACGGTGTTACTCGCAGTGTGACT<br>AAGCGTTCAGTCATGACGCTGGCTTACGGGTCCAAAGAGTTCGGCTTCCGTCAA<br>CAAGTGCTGGAAGATACCATTCAGCCAGCTATTGATTCCGGCAAGGGTCTGATG<br>TTCACTCAGCCGAATCAGGCTGCTGGATACATGGCTAAGCTGATTTGGGAATCT<br>GTGAGCGTGACGGTGGTAGCTGCGGTTGAAGCAATGAACTGGCTTAAGTCTGCT<br>GCTAAGCTGCTGGCTGCTGAGGTCAAAGATAAGAAGACTGGAGAGATTCTTCGC<br>AAGCGTTGCGCTGTGCATTGGGTAACTCCTGATGGTTTCCCTGTGTGGCAGGAA<br>TACAAGAAGCCTATTCAGACGCGCTTGAACCTGATGTTCCTCGGTCAGTTCCGC

			TTACAGCCTACCATTAACACCAACAAAGATAGCGAGATTGATGCACACAAACAG<br>GAGTCTGGTATCGCTCCTAACTTTGTACACAGCCAAGACGGTAGCCACCTTCGT<br>AAGACTGTAGTGTGGGCACACGAGAAGTACGGAATCGAATCTTTTGCACTGATT<br>CACGACTCCTTCGGTACCATTCCGGCTGACGCTGCGAACCTGTTCAAAGCAGTG<br>CGCGAAACTATGGTTGACACATATGAGTCTTGTGATGTACTGGCTGATTTCTAC<br>GACCAGTTCGCTGACCAGTTGCACGAGTCTCAATTGGACAAAATGCCAGCACTT<br>CCGGCTAAAGGTAACTTGAACCTCCGTGACATCTTAGAGTCGGACTTCGCGTTC<br>GCGTAA
	V	$T_7T\varphi$ Terminator	~~TACTAGAG~~CTGCTAACAAAGCCCGAAAGGAAGCTGAGTTGGCTGCTGCCACCGC<br>TGAGCAATAACTAGCATAACCCCTTGGGGCCTCTAAACGGGTCTTGAGGGGTTT<br>TTTGCTGAAAGGAGGAACTATATCCGGAT

[1] The final circuit can be generalized through seamless assembly in the order of their roman numerals. Then the circuits can be loaded into corresponding plasmids (Supplementary Table 1) by seamless continuation to the 3'-ends of each backbone. The same strategy could also be used for RPA circuit assembly of cross-species test (Supplementary Figure 10), follow the order of [pAR-(I-VII)]-[pBB-(I-V)] on the pABn, and [pAP-(I-VI)] on the pAPn.

[2] For all parts, non-functional regions (due to assembling scars) were shown with delete lines.

[3] The T_7 promoter library number followed by our previous work(2).

[4] The necessary 4 repeat sequences of gRNA processing region were shown with underlines(3). The others were 3 gRNA targets and corresponding locations in B node were indicated in bold.

[5] The terminator was composited with *rrnB* T1 terminator and T_7Te terminator.

Supplementary Table 3

Corresponding part substitutions for each perturbation experiment.

Perturb edges[1]	Replace regions	New Parts	Sequence (5' - 3')[2]	Related Figures
Non perturbations (RPA v1.0 circuit)				Figure 2B-C, Supplementary Figure 4A
B → B	pBB - I	pT$_7$m$_{22}$[3]	TAAACCGACTCACTATAGGGG~~TACTAGAG~~	Figure 2D, Figure 3A (middle), Supplementary Figure 4B
B → B	pBB - I	pT$_7$m$_{46}$[3]	GTGAACGACTCACTATAGGGG~~TACTAGAG~~	Figure 2E, Figure 3A (bottom), Supplementary Figure 4C
B → A	pAP - I	pT$_7$m$_{27}$[3]	TGATACGACTCACTATAGGGG	Figure 3B (middle), Figure 4A, Supplementary Figure 5A
B → A	pAP - I	pT$_7$m$_{45}$[3]	CTCTGCGACTCACTATAGGGG	Figure 3B (bottom), Supplementary Figure 5A
A ⊣ B	pAR - III	gRNA region[4]	GTCTAAGAACTTTAAATAATTTCTACTGTTGTAGATCCAAAGGTGGTCCGCTGCCGGTCTAAGAACTTTAAATAATTTCTACTGTTGTAGATAAATGGGAACGTGTTATGAAGTCTAAGAACTTTAAATAATTTCTACTGTTGTAGAT	Figure 3C (bottom), Supplementary Figure 5B
	pBB - II	5'-UTR region[4, 5]	AAAGTTA**CCAAAGGTGGTCCGCTGCCG**TTCGCTTGGGACATCCTGTCCCCGCAGTTCCAGTACGGTTCCAAAGCTTACGTTAAACACCCGGCTGACATCCCGGACTACCTGAAACTGTCCTTCCCGGAAGGTTTC**AAATGGGAACGTGTTATGAA**CTTCGAAGACGGTGGTGTTGTTACCGTTACCCAGGACTCCTCCCTGCAAGACGGTGAGTTCATCTACAAAGTTAAACTGCGTGGTACCAACTTCC~~tactagag~~agtacgtctgagcgtgatacccgctcactgaagatggcccggtagggccgaaacgtacctctacaaataattttgtttaa	Figure 3C (middle, bottom), Supplementary Figure 5B
Non-linear PFL	pAR – I	pR73 δ	GCCCGCCTTTTCTTTACCGGTGGTTGTGCTGTCGATTAGCCAACCGGGACAAATAGCCTGACATTACCTCTGGCGGTGATAATGGTTGCATGTACT	Supplementary Figure 9
	pAP – I			
	pBB – I			
	pBB - III	ϕ R73	ATGATGAGCGATAGTCCGCAGAAACTGGGTCGTAATGAATGGAATGCCTATATGGATAAAGTGAAAGCCAAAGA	

			TCGTGAAGCCTTTGCATTTGTGTTTCGTTTTTATGCA<br>CCGAAACTGAAACAGTTCGCCTATAAACATGTGGGTA<br>ATGAACAGGTTGCCATGGAAATGGTTCAAGAAACCAT<br>GGCCACCGTTTGGCAGAAAGCACATCTGTATGATGGT<br>AAAAAAAGCGCACTGAGCACCTGGATTTATACCATTA<br>TTCGCAACCTGTGCTTTGATCTGCTGCGTAAACAGAA<br>AGGTAAAGAACTGCATATCCACTCCGATGATATTTGG<br>CCGAGCGAATATTATCCGCCTGATATGGTTGATCACT<br>ATAGTCCGGAACAGGATATGCTGAAAGAACAGGTGGT<br>GAAATTTCTGGATATCCTGCCGAAAAATCAGCGTGAT<br>GTTCTGCAGGCAGTTTATCTGGAAGAACTGCCGCATC<br>AGCAGGTTGCAGAACTGTTTGATATTCCGCTGGGCAC<br>CGTTAAAAGCCGTCTGCGTCTGGCAGTTGAAAAACTG<br>CGTCATAGCATGCATACCGAACAGCTGTAA	
	pBB - V	RBS-mRFP1-$T_7T\varphi$ Terminator[6]	tactagagtctagatttaagaaggagatatacatATG<br>GCTTCCTCCGAAGACGTTATCAAAGAGTTCATGCGTT<br>TCAAAGTTCGTATGGAAGGTTCCGTTAACGGTCACGA<br>GTTCGAAATCGAAGGTGAAGGTGAAGGTCGTCCGTAC<br>GAAGGTACCCAGACCGCTAAACTGAAAGTTACCAAAG<br>GTGGTCCGCTGCCGTTCGCTTGGGACATCCTGTCCCC<br>GCAGTTCCAGTACGGTTCCAAAGCTTACGTTAAACAC<br>CCGGCTGACATCCCGGACTACCTGAAACTGTCCTTCC<br>CGGAAGGTTTCAAATGGGAACGTGTTATGAACTTCGA<br>AGACGGTGGTGTTGTTACCGTTACCCAGGACTCCTCC<br>CTGCAAGACGGTGAGTTCATCTACAAAGTTAAACTGC<br>GTGGTACCAACTTCCCGTCCGACGGTCCGGTTATGCA<br>GAAAAAAACCATGGGTTGGGAAGCTTCCACCGAACGT<br>ATGTACCCGGAAGACGGTGCTCTGAAAGGTGAAATCA<br>AAATGCGTCTGAAACTGAAAGACGGTGGTCACTACGA<br>CGCTGAAGTTAAAACCACCTACATGGCTAAAAAACCG<br>GTTCAGCTGCCGGGTGCTTACAAAACCGACATCAAAC<br>TGGACATCACCTCCCACAACGAAGACTACACCATCGT<br>TGAACAGTACGAACGTGCTGAAGGTCGTCACTCCACC<br>GGTGCTTAA~~tactagag~~ctgctaacaaagcccgaaag<br>gaagctgagttggctgctgccaccgctgagcaataac<br>tagcataaccccttggggcctctaaacgggtcttgag<br>gggtttttttgctgaaaggaggaactatatccggat	
PFL damage	pBB - I	BBa_ J23109	TTTACAGCTAGCTCAGTCCTAGGGACTGTGCTAGC~~TA~~<br>~~CTAGAG~~	Supplementary Figure 7B (left)
PFL damage	pBB - I	BBa_ J23105	TTTACGGCTAGCTCAGTCCTAGGTACTATGCTAGC~~TA~~<br>~~CTAGAG~~	Figure 3D, Supplementary Figure 7B (middle)
PFL	pBB - I	BBa_	TTGACAGCTAGCTCAGTCCTAGGTATTGTGCTAGC~~TA~~	Supplementary

damage		J23104	~~CTAGAG~~	Figure 7B (right), Supplementary Figure 8
NFL damage	pAP - (I - VI)	No Parts		Figure 3E

[1] The arrow indicates activation edge and the flat arrow indicates repression edge.

[2] For all parts, non-functional regions (due to assembling scars or other reasons) were shown with delete lines.

[3] The T_7 promoter library number followed by our previous work(2).

[4] The necessary 3 repeat sequences of gRNA processing region were shown with underlines(3). The others were 2 gRNA targets and corresponding locations in B node were indicated in bold.

[5] The Ribozyme-based insulator (original pBB - Ⅱ) was shown with lower-case letter.

[6] The mRFP1 CDS was indicated in capital letters.

Supplementary Table 4

Construction Details of other simple sub-circuits[1].

Experiments[2]	Back bones	Replace regions	New Parts	Sequence (5' - 3')[3]
T_7 RNAP Activation Ouput[4] (Figure 1D, Supplementary Figure 2)	pAR	I	pT_7m_1	TATAACGACTCACTATAGGGG
			pT_7m_3	GGATACGACTCACTATAGGGG
			pT_7m_4	TAATACGACTCAGTCAAGGGG
			pT_7m_5	GTGCACGACTCACTATAGGGG
			pT_7m_6	TAATACGACTCACACTCGGGG
		II	No parts	
In → A (Supplementary Figure 3A)	pAR	/	/	/
	pBB	/	/	/
A ⊣ B (Supplementary Figure 3C)	pAR	I	T_5 Promoter	TCATAAAAAATTTATTTGCTTTGTGGCGGATAACAATTATAATA~~GATTC~~
	pAP	I	T_5 Promoter	TCATAAAAAATTTATTTGCTTTGTGGCGGATAACAATTATAATA~~GATTC~~
	pBB	I	BBa_ J23104	TTGACAGCTAGCTCAGTCCTAGGTATTGTGCTAGC~~TACTAGAG~~
		V (*)	RBS-mRFP1-$T_7T\varphi$ Terminator[5]	tactagagtctagatttaagaaggagatatacatATGGCTTCCTCCGAAGACGTTATCAAAGAGTTCATGCGTTTCAAAGTTCGTATGGAAGGTTCCGTTAACGGTCACGAGTTCGAAATCGAAGGTGAAGGTGAAGGTCGTCCGTACGAAGGTACCCAGACCGCTAAACTGAAAGTTACCAAAGGTGGTCCGCTGCCGTTCGCTTGGGACATCCTGTCCCCGCAGTTCCAGTACGGTTCCAAAGCTTACGTTAAACACCCGGCTGACATCCCGGACTACCTGAAACTGTCCTTCCCGGAAGGTTTCAAATGGGAACGTGTTATGAACTTCGAAGACGGTGGTGTTGTTACCGTTACCCAGGACTCCTCCCTGCAAGACGGTGAGTTCATCTACAAAGTTAAACTGCGTGGTACCAACTTCCCGTCCGACGGTCCGGTTATGCAGAAAAAAACCATGGGTTGGGAAGCTTCCACCGAACGTATGTACCCGGAAGACGGTGCTCTGAAAGGTGAAATCAAAATGCGTCTGAAACTGAAAGACGGTGGTCACTACGACGCTGAAGTTAAAACCACCTACATGGCTAAAAAACCGGTTCAGCTGCCGGGTGCTTACAAAACCGACATCAAACTGGACATCACCTCCCACAACGAAGACTACACCATCGTTGAACAGTACGAACGTGCTGAAGGTCGTCACTCCACCGGTGCTTAA~~tactagag~~ctgctaacaaagcccgaaaggaagctgagttggctgctgccaccgctgagcaataactagcataaccccttggggcctctaaacgggtcttgaggggttttttgctgaaaggaggaactatatccggat

B → A (Supplementary Figure 3B)	pAR	/	/	/
	pAP	I - VI	No parts	
	pBB	I	Tac promoter[6]	TGTTGACAATTAATCATCGGCTCGTATAATGTGTGGAATTGTGAGCGCTCACAATT
		V	Same as (*)	

[1] All sub-circuits were constructed based on the RPA v1.0 unless otherwise specified.

[2] The arrow indicates activation edge and the flat arrow indicates repression edge.

[3] For all parts, non-functional regions (due to assembling scars or other reasons) were shown with delete lines.

[4] The table only summarized the reporter cassette details of the RNAP activation test.

[5] The mRFP1 CDS was indicated in capital letters.

[6] The underlined regions show the -35, -10 regions and *lacO* sequence of pTac.

Details of Modeling

We constructed a more detailed biophysical dynamic model, and try to fit parameters in this model and validate its effectiveness by compare its prediction values with corresponding experimental data.

The detailed model is:

$$\frac{d[A]}{dt} = \alpha_A \left(\frac{1}{1 + \left(\frac{K_{TF_{tot}}}{1 + {[Input]}/{K_{IR}}} \right)^{n_{RA}}} + \beta_{RA} \right) \left(\frac{[B]^{n_B}}{{K_{BA}}^{n_B} + [B]^{n_B}} + \beta_{BA} \right) - \gamma_A [A]$$

$$\frac{d[B]}{dt} = \alpha_B \left(\frac{1}{1 + \left(\frac{[A]}{K_{AB}} \right)^{n_{AB}}} + \beta_{AB} \right) \left(\frac{[B]^{n_B}}{{K_{BB}}^{n_B} + [B]^{n_B}} + \beta_{BB} \right) - \gamma_B [B]$$

The biophysical model has several detailed changes compared with the original theoretical model(4). For the induction from Input to A node, it is a de-repression process that Cumate inducer affect the transcription repressor CymR, thus the function is re-written into a repression form, using two new parameters, $K_{TF_{tot}}$ and n_{RA} to represent the transcriptional regulation of CymR. The Inducer effect is represented with K_{IR} parameter, which describes the affinity between Cumate and CymR. For all regulation functions, a constant item β_{ij} is added, representing the leakage expression level due to the context effect, which is prevalent in genetic circuits. Because the same activation part was used for both activation from B node to A node and self-activation of B node, the Hill coefficient n_{BA} and n_{BB} in the main text were merged as n_B.

The parameter fitting results are summarized in Supplementary Table 5.

Supplementary Table 5

Details of Parameter Values.

Parameters[1]	Meanings	Values	Units[3]	Related Figures
α_A	Maximal expression level of A node	653.04	A.U./hr	Figure 2C-E, Supplementary Figure 4
α_B	Maximal expression level of B node	3.11e6	A.U./hr	
β_{RA}	Repression leakage scale from CymR to A node	0.14	/	

β_{BA}	Activation leakage scale from B node to A node	9.97e-7	/	
β_{AB}	Repression leakage scale from A node to B node	0	/	
β_{BB}	auto-activation leakage scale of B node	0.037	/	
γ_A	dilution and degradation rate constant of A node	1.78	hr^{-1}	
γ_B	dilution and degradation rate constant of B node	0.70	hr^{-1}	
n_{RA}	Hill coefficient of Transcription Factor	2.34	/	
n_B	Hill coefficient of B node	1.30	/	
n_{AB}	Hill coefficient of A node	3.20	/	
$K_{TF_{tot}}$	CymR affinity constant	2603.85	/	
K_{IR}	Cumate affinity constant	1.28e-8	mol/L	
K_{BA}	Hill constant from B node to A node	36.41	A.U.	
K_{AB}	Hill constant from A node to B node	0.51	A.U.	
${K_{BB}}^2$	Hill constant of B node itself for pT_7m_3	4006.90	A.U.	Figure 2C, Supplementary Figure 4A
	Hill constant of B node itself for pT_7m_{22}	2093.89	A.U.	Figure 2D, Supplementary Figure 4B
	Hill constant of B node itself for pT_7m_{46}	15910.97	A.U.	Figure 2E, Supplementary Figure 4C

[1] Parameters summarized here are fitting results using RPA v1.0 (Figure 2C, Supplementary Figure 4A) and are used for predicting except K_{BB}.

[2] The Hill constants of pT_7m_{22} and pT_7m_{46} are used for predicting. The strength ratio between these promoters follow the relationships in (2).

[3] A.U. is the arbitrary unit of the fluorescence in single cells of flow cytometer.

Reference

1. Lou, C., Stanton, B., Chen, Y.-J., Munsky, B. and Voigt, C.A. (2012) Ribozyme-based insulator parts buffer synthetic circuits from genetic context. *Nature Biotechnology*, **30**, 1137-1142.

2. Zong, Y., Zhang, H.M., Lyu, C., Ji, X., Hou, J., Guo, X., Ouyang, Q. and Lou, C. (2017) Insulated transcriptional elements enable precise design of genetic circuits. *Nat Commun*, **8**.

3. Miao, C., Zhao, H., Qian, L. and Lou, C. (2019) Systematically investigating the key features of the DNase deactivated Cpf1 for tunable transcription regulation in prokaryotic cells. *Synthetic and Systems Biotechnology*, **4**, 1-9.

4. Shi, W., Ma, W., Xiong, L., Zhang, M. and Tang, C. (2017) Adaptation with transcriptional regulation. *Scientific Reports*, **7**, 42648.
